# Weight-of-Evidence Strategies to Mitigate the Influence of Messages of Science Denialism in Public Discussions

**DOI:** 10.5334/joc.125

**Published:** 2020-10-01

**Authors:** Philipp Schmid, Marius Schwarzer, Cornelia Betsch

**Affiliations:** 1 CEREB – Center of Empirical Research in Economics and Behavioral Sciences, University of Erfurt, Erfurt, DE; 2 Department of Psychology, University of Erfurt, Erfurt, DE; 3 Department of Media and Communication Science, University of Erfurt, Erfurt, DE

**Keywords:** false-balance effect, science denialism, forewarning, multiple-source effect, rebuttal, weight of evidence, vaccination

## Abstract

In mass media, the positions of science deniers and scientific-consensus advocates are repeatedly presented in a balanced manner. This false balance increases the spread of misinformation under the guise of objectivity. Weight-of-evidence strategies are an alternative, in which journalists lend weight to each position that is equivalent to the amount of evidence that supports the position. In public discussions, journalists can invite more advocates of scientific consensuses than science deniers (outnumbering) or they can employ warnings about the false-balance effect prior to the discussions (forewarning). In three pre-registered laboratory experiments, we tested the efficacy of outnumbering and forewarning as weight-of-evidence strategies to mitigate science deniers’ influence on individuals’ attitudes towards vaccination and their intention to vaccinate. We explored whether advocates’ responses to science deniers (rebuttal) and audiences’ issue involvement moderate the efficacy of these strategies. A total of *N* = 887 individuals indicated their attitudes towards vaccination and their intention to vaccinate before and after watching a television (TV) discussion. The presence and absence of forewarning, outnumbering and rebuttal were manipulated between subjects; participants also indicated their individual issue involvement. We obtained no evidence that outnumbering mitigates damage from denialism, even when advocates served as multiple sources. However, forewarning about the false-balance effect mitigated deniers’ negative effects. Moreover, the protective effect was independent of rebuttal and issue involvement. Thus, forewarnings can serve as an effective, economic and theory-driven strategy to counter science denialism in public discussions, at least for highly educated individuals such as university students.

Objectivity is a maxim in journalism that aims to reduce bias in media reports by eliminating journalists’ subjective interpretations (Boudana, 2016; Schudson, 2001). A frequently applied strategy to achieve objectivity is to balance media reports by contrasting two opposing positions on the same issue, leaving it to the audience to weigh the positions and draw conclusions about the subject matter. However, balancing can reduce bias only if both opposing positions are supported by an equal amount of evidence (Boudana, 2016; Dearing, 1995). If this assumption is violated, balancing can turn into a source of bias itself. For example, a wide consensus in scientific communities exists that human-made climate change is happening (Cook et al., 2016), that vaccines are beneficial (PRC, 2015) and that humans have evolved over time (PRC, 2009). However, some individuals reject these scientific consensuses (Hornsey & Fielding, 2017; Lewandowsky & Oberauer, 2016) and ‘employ rhetorical arguments to give the appearance of a legitimate debate where there is none’ (Diethelm & McKee, 2009, p. 2). That is, they represent a form of pseudoscience known as *science denialism* (Hansson, 2017). In mass media, science deniers’ views and positions within scientific consensuses repeatedly are presented in a balanced fashion (climate change: Petersen, Vincent & Westerling, 2019; vaccination: Clarke, 2008; evolution: Mooney & Nisbet, 2005). In these instances, journalists ignore scientific consensuses’ greater weight of evidence and apply a biased 50/50 weight to the presentation of contrasting positions, that is, they apply *false balance* (Brüggemann & Engesser, 2017; Dixon & Clarke, 2013; Koehler, 2016). Extant research shows that falsely balanced reports can distort positive attitudes towards behaviours that science favours and decrease individuals’ intentions to perform these behaviours (Corbett & Durfee, 2004; Dixon & Clarke, 2013). Thus, false balance increases damage from science denialism messages under the guise of objectivity.

The issue of false balance is especially prevalent in public discussions that are broadcasted on TV or radio. These discussions often are designed to highlight or induce conflict (Rubin & Step, 1997). Producers often invite people with opposing opinions or perspectives to challenge invited experts’ opinions on social, political or personal issues. Scientific evidence thus stands one-on-one with personal opinions, statistical numbers next to emotional narratives (Livingstone & Lunt, 1994). To avoid potential damage to audiences from such falsely balanced public discussions (Dixon & Clarke, 2013), journalists either could refrain from broadcasting them or alter the environment in favour of evidence-based perspectives in discussions (Clarke, McKeever, Holton & Dixon, 2015; Dunwoody, 2005; Kohl et al., 2016). Here, we focus on the latter because we argue that the issue of false balance is not rooted in the very existence of an unscientific perspective, but in the inappropriate weight that this perspective receives in mass media (Boudana, 2016; Dearing, 1995). Thus, in the remainder of this paper, we will discuss several weighting strategies that journalists could use to support the voice of science while maintaining the freedom to broadcast public discussions that involve opposite opinions and contradictory scientific views, that is, maintain democratic discourse.

## Weight-of-evidence strategies

An alternative to falsely balanced discussions, which potentially can alter the environment in favour of evidence-based perspectives, is weight-of-evidence reporting (Dunwoody, 2005; Kohl et al., 2016). A weight-of-evidence strategy ‘calls on journalists not to determine what’s true, but instead to find out where the bulk of evidence and expert thought lies on the truth continuum and then communicate that to the audiences’ (Dunwoody, 2005, p. 90). Thus, weight-of-evidence strategies neither overestimate positions that are backed up with little evidence, nor do they neglect the existence of contrasting positions (Kohl et al., 2016). Instead, weight-of-evidence strategies provide each position in a public discussion with a weight corresponding to the amount of evidence that supports the position. Previous research shows that weight-of-evidence strategies in newspaper articles can mitigate damage to the audience’s attitudinal beliefs from misleading reports (Clarke, Dixon, Holton & McKeever, 2015; Clarke, McKeever, et al., 2015; Kohl et al., 2016). Given these promising findings, it is now necessary to explore how weight-of-evidence strategies can be best applied in public discussions about science.

## Outnumbering as a weight-of-evidence strategy

A variety of cues can be used as weights of evidence in public discussions. For instance, a journalist can counter false balance at a public forum by inviting more advocates for science than deniers, that is, the relative number of guests serves as weight of evidence. We refer to this strategy as *outnumbering*. Extant research in psychology supports the efficacy of such a strategy for two reasons. First, the number of discussants who are invited to represent each position may serve as a social cue (Wood, 2000). Psychological research repeatedly has proposed and demonstrated that individuals tend to align their own judgements with the majority opinion (Bond & Smith, 1996; Cialdini & Goldstein, 2004; McDonald & Crandall, 2015). Individuals either follow majorities to express conformity with others, or they use majorities as informational evidence of facts (McDonald & Crandall, 2015; Wood, 2000). In line with research on scientific consensus (Lewandowsky, Gignac & Vaughan, 2012; van der Linden, Clarke, & Maibach, 2015), using majorities as informational evidence seems especially reasonable when the majority comprises experts in the field under discussion.

Second, the number of discussants who are invited to represent each position can cause a multiple-source effect. Several studies have shown that arguments are more persuasive when presented by multiple sources rather than a single source (Harkins & Petty, 1987; Petty, Harkins & Williams, 1980). It has been suggested that this effect occurs because individuals invest their limited cognitive resources economically, that is, they prefer to process the most worthy information. Multiple sources are perceived as independent pools of knowledge that are likely to represent a wide variety of perspectives, while a single source’s perspective is likely to be known after the first argument (Harkins & Petty, 1987). Thus, information from multiple sources ‘is more worthy of diligent consideration than information from only one perspective’ (Harkins & Petty, 1987, p. 267).

Due to the consistent theoretical and empirical persuasive advantage of having multiple discussants, we expect that the relative number of guests representing a certain position can serve as a weight-of-evidence strategy in a public discussion. Therefore, the *outnumbering hypothesis* predicts that when advocates for science outnumber, rather than balance, science deniers in a public discussion, damage from denial will be mitigated. This mitigation will be achieved either due to the fact that the relative number of advocates serves as a social cue (Experiments 1–3), or due to an additional multiple-source effect (Experiment 3).

## Forewarning as a weight-of-evidence strategy

Another weight-of-evidence strategy is to warn the audience prior to the discussion. In line with inoculation theory (McGuire, 1961a, 1961b), individuals can activate their own ‘immune responses’ against persuasive attempts prior to the persuasion episode when perceiving a threat from being the target of inappropriate persuasion. This pre-activation can reduce the biasing effect of misinformation, including science denialism messages (Cook, Lewandowsky & Ecker, 2017; Roozenbeek & van der Linden, 2019). A common psychological intervention that uses prior information to apply weight to subsequent information is forewarning (McGuire & Papageorgis, 1962; Quinn & Wood, 2003). For example, being warned about a source’s persuasive intent before it is accessed can decrease the weight of the information that the source provides, thereby reducing its influence (Quinn & Wood, 2003). Due to falsely balanced public discussions’ biased nature, we expect that forewarning about the false-balance effect can serve as a weight-of-evidence strategy in favour of the scientific position. Thus, the *forewarning hypothesis* expects that when individuals read a general explanation of the false-balance effect, denial messages’ damage will be mitigated compared with a control group.

## Issue involvement and rebuttal as moderators

Some extant findings challenge the idea that weight-of-evidence strategies are a universal approach to counter science denialism in public discussions. First, forewarnings are found to be more effective with highly involved audiences, but also have a greater chance of backfiring with these specific audiences (Albarracín & Handley, 2011; Quinn & Wood, 2003). Moreover, outnumbering can be classified as a peripheral cue. Dual process models suggest that peripheral cues should be more effective with less-involved audiences because peripheral cues do not require as much motivation as a central message feature to be persuasive (Petty & Cacioppo, 1986). On the contrary, the Unimodel argues that the required motivation increases with increasing complexity of a message feature and not just because a feature happens to be peripheral or central (Kruglanski & Thompson, 1999). Given the mixed assumptions, the *involvement-as-moderator research question* explores whether and how the audience’s issue involvement will moderate weight-of-evidence strategies’ efficacy.

Second, the two outlined weight-of-evidence strategies’ efficacy may depend on whether an advocate responds to the denier’s claim with a counter message, that is, whether or not a rebuttal is present. If science deniers present misinformation, and advocates respond with rebuttal messages (Schmid & Betsch, 2019), then weight-of-evidence strategies can decrease the misinformation’s persuasiveness and/or increase the rebuttal’s persuasiveness. In the absence of a rebuttal, weight-of-evidence strategies only can decrease the persuasiveness of deniers’ misinformation. In a public discussion, a rebuttal may be absent if the science advocate is not given the chance to respond to the denier, he or she is not trained in rebuttals or he or she does not feel confident enough to demand speaking time (WHO, 2016). Thus, given the lack of previous research on the topic, the *rebuttal-as-moderator research question* explores whether and how weight-of-evidence strategies’ efficacy will depend on rebuttal messages’ presence. We refer to the failure to deliver a rebuttal as *advocate silent*.

## Overview

**Figure 1:**
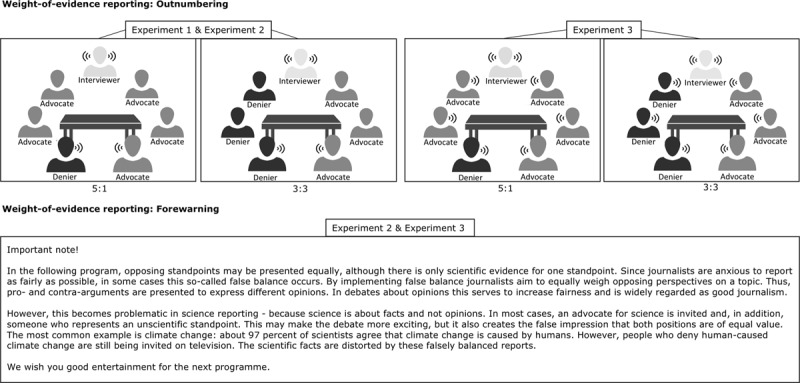
**Weight-of-evidence strategies in public discussions.** The displayed materials represent the weight-of-evidence strategies *outnumbering* and *forewarning* as used in the respective experiments. Following the social-cue mechanism of outnumbering, the number of science advocates and deniers varied between conditions (3:3 vs. 5:1) in all experiments. Following the additional multiple-source mechanism, all guests in the discussion contributed as speakers in Experiment 3. The presented forewarning text is a translated version of the original German forewarning used in Experiments 2 and 3.

In three preregistered laboratory experiments, we tested the efficacy of outnumbering (Experiments 1–3) and forewarning (Experiments 2 and 3) to counter the damage from science denialism in public discussions (Figure 1). We further explored whether these strategies’ efficacy depends on the audience’s issue involvement and on advocates’ successful delivery of rebuttals. All experiments focussed on public discussions about vaccination as a content domain. Vaccination is a behaviour favoured by science that has been addressed repeatedly through science denialism and falsely balanced discussions (Dixon & Clarke, 2013). Thus, vaccination is an appropriate testing ground for interventions to counter misinformation. All tested interventions aim to mitigate damage from science deniers to the public’s acceptance of science. Thus, samples were not drawn from the population of science deniers as they have a low willingness to change their mind compared to the general public, and an investigation of the effectiveness of weight-of-evidence strategies in this population would likely fail (WHO, 2016). All studies used Schmid and Betsch’s (2019) research scenario, in which participants read information about the fictitious disease dysomeria and learned that vaccination against the disease was available and recommended. Subsequently, participants watched a mock TV discussion between vaccine deniers and advocates for science, in which relevant independent variables were manipulated. Participants indicated their attitudes towards vaccination and the intention to get vaccinated against the fictitious disease before and after the discussion. The strategies’ efficacy was judged based on how strongly deniers changed previous attitudes and intention. As described under the rebuttal as the moderator’s research question, weight-of-evidence strategies could be effective because they decrease the persuasiveness of deniers’ messages and/or increase the persuasiveness of the rebuttal message by providing each message with a weight equivalent to the amount of evidence that supports the position. Following this rationale, failure to detect any weight-of-evidence strategies’ effects may be due to mere inefficacy of either the deniers’ message or the advocates’ rebuttal message in producing any persuasive effect. Thus, replicating the damaging effect from denial messages and the mitigating effect from rebuttal messages from Schmid and Betsch (2019) indicated successful manipulation in all subsequent experiments.

## Experiment 1

In this experiment, we tested the outnumbering hypothesis by assessing whether a relatively higher number of advocates for science compared with deniers effectively reduces damage from science denialism. Experiment 1 uses the relative number of advocates present as a social cue, that is, while the relative number of advocates and deniers varies, only one person per group serves as a speaker (Figure 1). In addition, we examined involvement and rebuttal-as-moderator research questions for the outnumbering strategy, and we preregistered the experiment on aspredicted.org: http://aspredicted.org/blind.php?x=jp7az5.

### Method

#### Participants and design

A-priori power analyses using G*Power (Faul, Erdfelder, Buchner & Lang, 2009) revealed a minimum sample size of *N* = 100 to detect an interaction effect of *d* = 0.34 with a power of .80 in a mixed within-between ANOVA (four groups, two measurements, α = .05). The chosen effect size of the power analysis was informed by effect sizes of previous research on the false-balance effect (Dixon & Clarke, 2013). Altogether, 101 psychology students at the University of Erfurt participated in this lab experiment in exchange for course credit. Participants received the invitation to participate via a mailing list, and each participant was assigned randomly to one of four conditions, resulting from the 2 (rebuttal vs. advocate silent; between subjects) × 2 (outnumbering 5:1 vs. equal proportion of guests 3:3; between subjects) × 2 (measurement before vs. after the debate; within subjects) mixed design.

#### Materials and Procedure

All materials were presented on a computer screen (Figure 1). Participants watched a mock TV discussion as part of a fictitious scenario. The TV discussion comprised videos that were designed specifically for the experiments. The videos depicted a public discussion, and the critical stimulus materials were voice recordings that Schmid and Betsch (2019) already have used. The videos indicated the number of participating guests and highlighted the current speaker, but contained few other details to minimise distraction and potential confounders. Figure 1 presents screenshots of the videos, all of which can be accessed at the Open Science Framework (Schmid, Schwarzer & Betsch, 2019). Depending on condition, science advocates in the videos either outnumbered the deniers (5:1) or were equal in number (3:3). The proportions of advocates and deniers that participated in the TV discussion were highlighted with different colours. An interviewer introduced the speakers for the TV discussion, during which one vaccine denier delivered the same two messages in all conditions. In these messages, the denier questioned the safety of vaccination against dysomeria, as well as health authorities’ trustworthiness, by using the rhetorical techniques of impossible expectation and conspiracy theories. Depending on condition, one science advocate either refuted the denier’s messages, or all advocates remained silent. Only one denier and one advocate delivered the messages, independent of the proportion of guests. Denial and rebuttal messages resembled those that Schmid and Betsch (2019) used in Experiment 1, where they are described in detail. A fully translated script of messages used in this study is available under Supplementary Method in the Supplementary Information. After the second measurement of primary outcomes, individuals’ issue involvement, speakers’ perceived credibility, additional control variables and demographic data were assessed.

#### Measures

Participants indicated their attitudes towards vaccination and intention to get vaccinated before and after the TV discussion (Table 1). The primary measures were identical to those that Schmid and Betsch (2019) used. In addition, we added a single item on individuals’ willingness to donate to a fictitious anti-vaccination campaign. Willingness to donate was added for explorative purposes only and was dropped for Experiments 2 and 3. After completing the dependent measures, participants answered a single attention question about the discussion’s content, individuals’ issue involvement, speakers’ perceived credibility, additional control variables and demographic information (Supplementary Table 1).

**Table 1:** **Overview of outcome measures.** Reliability of multiple-item scales is indicated by Cronbach’s alpha; numbers behind alphas relate to the respective experiments. All outcome measures were converted into percentages of maximum possible scores of the original scales (POMP), with higher values indicating a more positive attitude, greater confidence in vaccination and stronger intention.

Construct	Incl.	Scale type	Wording	Source

attitude towards vaccination	Exp. 1–3	mean score of 5-point rating scales (α1pre = .72; α1post = .85; α2pre = .81; α2post = .86; α3pre = .84; α3post = .88)	Please indicate how much you agree with the following statements. 1. Vaccinating against dysomeria is necessary. 2. Vaccinating against dysomeria is a good idea. 3. Vaccinating against dysomeria is beneficial. (1 = I strongly disagree, 5 = I strongly agree)	(Schmid & Betsch, 2019)
intention to get vaccinated	Exp. 1–3	visual analog scale	If you had the opportunity to get vaccinated against dysomeria, what would you do? (1 = I will definitely not get vaccinated, 100 = I will definitely get vaccinated)	(Schmid & Betsch, 2019)
confidence in vaccination	Exp. 2–3	mean score of 5-point rating scales (α2pre = .71; α2post = .83; α3pre = .77; α3post = .85)	Please indicate how much you agree with the following statements. 1. Vaccination against dysomeria is effective. 2. I am completely confident that the vaccine against dysomeria is safe. 3. Regarding the vaccine against dysomeria, I am confident that public authorities decide in the best interest of the community. (1 = I strongly disagree, 5 = I strongly agree)	(Betsch et al., 2018)

#### Data analyses

For all experiments, we used repeated-measures ANOVA models in IBM SPSS 25 for hypothesis testing. All repeated-measures ANOVA models were conducted as preregistered in all experiments. When adding a continuous moderator for the involvement-as-moderator research question, we used linear models on change scores in R. The involvement-as-moderator research question and the associated analyses were not preregistered. Thus, the research question and the linear models are described as explorative throughout the manuscript. We used type 2 sum of squares and a 0.05 significance level for all models. All outcome measures were transformed into percentages of maximum possible scores from the original scales (POMP = [(observed score – minimum possible score)/(maximum possible score – minimum possible score)] × 100; P. Cohen, Cohen, Aiken & West, 1999). The linear transformation of the original scores into POMP scores allows for easy interpretation of outcome measures as all scores range from 0 to 100 after transformation. An increase of one unit on a POMP scale can be translated to an increase of 1% of the maximum possible score of the original scale. For example, an increase in the attitude towards vaccination by 20 units (%) of the POMP scale would translate to an increase of one point on the original five-point scale. Positive POMP values of change scores indicate the percent increase in attitudes and intentions and negative POMP values of change scores indicate a percent decrease in attitudes and intentions in all figures.

### Results

Eighty percent of the full sample was female, with a mean age of 20.71 (*SD*_age_ = 2.71) for all participants. All participants correctly identified the hypothetical debate’s content; thus, no participant was excluded from the analyses. No evidence of differences between conditions in prior attitudes towards vaccination or prior intention to get vaccinated was found (all *p*s ≥ .150 in 2 × 2 ANOVAs). The Supplementary Information contains detailed descriptive data on all experiments (Supplementary Method).

**Figure 2:**
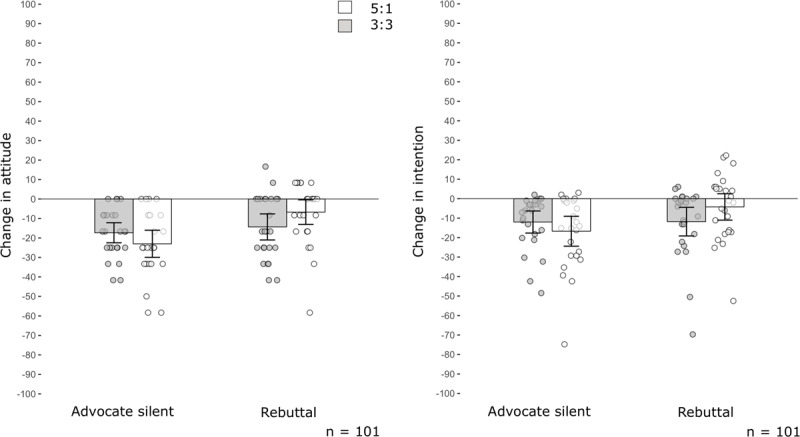
**Effects from outnumbering on damage from science denialism in public discussions in Experiment 1.** The results reveal that outnumbering mitigated the damage from denialism only if a rebuttal was delivered. The y-axes represent mean changes in attitude (left graph) and in intention (right graph) in POMP values. Descriptive data are provided in Supplementary Tables 2–4. The x-axes represent experimental conditions. Error bars represent 95% CIs. Dots indicate changes in individual participants’ attitudes and intentions.

Tables 2 and 3 and Figure 2 present the results from the 2 × 2 × 2 repeated-measures ANOVA models for individuals’ attitudes and intention. Watching the public debate significantly damaged individuals’ attitudes towards vaccination and their intention to get vaccinated, but the rebuttal successfully mitigated this damage. The mitigating effect was only marginally significant on intention to get vaccinated. These results indicate successful manipulation and replicate previous findings (Schmid & Betsch, 2019).

**Table 2:** Weight-of-evidence strategies’ effects on changes in attitude. The results presented in Tables 2–4 are based on a 2 (rebuttal vs. advocate silent; between subjects) × 2 (outnumbering 5:1 vs. equal proportion of discussants 3:3; between subjects) × 2 (forewarning vs. no forewarning) × 2 (measurement before vs. after the debate; within subjects) repeated-measures ANOVA (Type II sum of squares). Significant effects are shown in boldface for the significance level of *p* < 0.05.

Attitude	Experiment 1 n = 101			Experiment 2 n = 390			Experiment 3 n = 396		
	
Effects	*F*	*p*	η^2^_*p*_	*F*	*p*	η^2^_*p*_	*F*	*p*	η^2^_*p*_

Time	**99.16**	**<.001**	**.506**	**165.42**	**<.001**	**.302**	**132.11**	**<.001**	**.254**
Rebuttal × Time	**10.01**	**.002**	**.094**	**24.13**	**<.001**	**.059**	**50.91**	**<.001**	**.116**
Outnumbering × Time	0.11	.737	.001	0.27	.605	.001	2.10	.148	.005
Forewarning × Time	—	—	—	**7.52**	**.006**	**.019**	1.11	.293	.003
Rebuttal × Outnumbering × Time	**4.68**	**.033**	**.046**	0.02	.883	<.001	2.83	.093	.007
Rebuttal × Forewarning × Time	—	—	—	0.47	.495	.001	0.16	.687	<.001
Outnumbering × Forewarning × Time	—	—	—	1.40	.239	.004	0.02	.886	<.001
Rebuttal × Outnumbering × Forewarning × Time	—	—	—	0.54	.462	.001	3.37	.067	.009

**Table 3:** **Weight-of-evidence strategies’ effects on changes in intention.** Significant effects are shown in boldface for the significance level of *p* < 0.05.

Intention	Experiment 1 n = 101			Experiment 2 n = 390			Experiment 3 n = 396		
	
Effects	*F*	*p*	η^2^_*p*_	*F*	*p*	η^2^_*p*_	*F*	*p*	η^2^_*p*_

Time	**43.98**	**<.001**	**.312**	**156.64**	**<.001**	**.291**	**92.10**	**<.001**	**.192**
Rebuttal × Time	3.65	.059	.036	**32.37**	**<.001**	**.078**	**57.58**	**<.001**	**.129**
Outnumbering × Time	0.19	.661	.002	0.15	.703	<.001	2.44	.119	.006
Forewarning × Time	—	—	—	**14.75**	**<.001**	**.037**	**6.92**	**.009**	**.018**
Rebuttal × Outnumbering × Time	3.36	.070	.033	0.03	.868	<.001	0.03	.865	<.001
Rebuttal × Forewarning × Time	—	—	—	<0.01	.948	<.001	0.60	.441	.002
Outnumbering × Forewarning × Time	—	—	—	0.03	.864	<.001	1.11	.293	.003
Rebuttal × Outnumbering × Forewarning × Time	—	—	—	0.07	.790	<.001	0.25	.615	.001

However, contrary to the expectations on the outnumbering hypothesis, we obtained no evidence that the presence of a higher proportion of science advocates at the debate mitigated deniers’ influence on individuals’ attitudes or intention; interaction effects from outnumbering with time on attitude: *F*(1, 97) = 0.11, *p* = .737, η^2^*_p_* = .001, intention: *F*(1, 97) = 0.19, *p* = .661, η^2^*_p_* = .002. We repeated confirmatory analyses with preregistered control variables, and the pattern of results did not differ (Supplementary Tables 5 and 6).

Further examination of the rebuttal-as-moderator research question (Table 2; Figure 2) revealed a significant interaction effect from outnumbering, rebuttal and time on individuals’ attitudes, *F*(1, 97) = 4.68, *p* = .033, η^2^*_p_* = .046. That is, inviting more advocates than deniers marginally reduced damage from deniers when a rebuttal was delivered, *F*(1, 97) = 3.10, *p* = .081, though this trend was reversed when the advocate remained silent, *F*(1, 97) = 1.69, *p* = .196. The interaction effect was only marginally significant concerning individuals’ intention (Table 3).

Additional explorative analyses revealed no evidence that the outnumbering strategy’s effects depend on the audience’s issue involvement (Supplementary Table 7). Furthermore, we found no evidence that the willingness to donate to a fictitious initiative that supports anti-vaccination campaigns differed between either the rebuttal or outnumbering conditions (χ^2^s < 1).

### Discussion

Contrary to expectations on the outnumbering hypothesis, we found no evidence that inviting a greater number of advocates significantly mitigates science denialism’s influence on the audience. However, in exploring the rebuttal-as-moderator research question, we found tentative evidence that outnumbering may be an effective weight-of-evidence strategy after all, but only if the advocate successfully delivers a rebuttal. Thus, in the following experiment, we converted our initial rebuttal-as-moderator research question into the preregistered *rebuttal-as-moderator hypothesis*. Furthermore, we tested the efficacy of forewarning as a new weight-of-evidence strategy.

## Experiment 2

In this experiment, we tested the rebuttal-as-moderator hypothesis and the forewarning hypothesis. Thus, we expected that forewarning would decrease denialism messages’ damage and that outnumbering is only effective when a rebuttal takes place and when no forewarning was implemented. The latter restriction of the rebuttal-as-moderator hypothesis was included because the forewarning was assumed to knock out any false-balance effect. In addition, we explored the rebuttal-as-moderator research question for forewarning and the involvement-as-moderator research question for both weight-of-evidence strategies. We preregistered the experiment on aspredicted.org: http://aspredicted.org/blind.php?x=ie3es8. However, the forewarning hypothesis was not preregistered.

### Method

The experimental setup was almost identical to Experiment 1, with deviations described below.

#### Participants and design

A-priori power analyses using G*Power (Faul et al., 2009) revealed a minimum sample size of *N* = 368 to detect an interaction effect (minimum effect size of interest of *d* = 0.20) with a power of .80 in a mixed within-between ANOVA (eight groups, two measurements, α = .05). The effect size of the power analysis was chosen to detect conventionally small effect sizes (J. Cohen, 1977). Altogether, 390 undergraduate students at the University of Erfurt participated in this lab experiment. Subjects volunteered to participate during their university welcome week and did not receive any incentives. Each subject was assigned randomly to one of eight conditions, resulting from a 2 (rebuttal vs. advocate silent; between subjects) × 2 (outnumbering 5:1 vs. equal proportion of discussants 3:3; between subjects) × 2 (forewarning vs. no forewarning) × 2 (measurement before vs. after the debate; within subjects) mixed design. In contrast to Experiment 1, we did not preregister any exclusion criteria, but planned to stratify results based on individuals’ attention.

#### Materials and procedure

The forewarning was implemented within the scenario. The participants imagined to be users of an online media centre that broadcasted the TV discussion. The control group read a neutral text about data protection for online users who access a media centre (Supplementary Method) while the experimental group read an explanatory text about false balance in media reports (Figure 1).

#### Measures

In addition to the measures in Experiment 1 (Table 1, Supplementary Table 1), participants indicated their confidence in the fictitious vaccination before and after the TV discussion. Confidence assesses one’s beliefs in vaccination’s safety and efficacy, as well as perceived trustworthiness of the institutions that deliver them (Betsch et al., 2018). Thus, it represents the audience’s specific attitudinal beliefs towards the topics that science deniers target, that is, vaccination safety and trust in institutions. As an additional attention check, we asked participants in an open format about the number of advocates and deniers who were present during the discussion (Supplementary Table 1).

### Results

Seventy-seven percent of the full sample was female, with a mean age of 19.96 (*SD*_age_ = 2.26) for all participants. 99.5% correctly answered the question about the debate’s content, with 80% recalling the exact number of advocates and 82.3% recalling the exact number of deniers who were present at the debate. Thus, we repeated the primary analyses with a sample containing only those participants who recalled the correct information, with differences in the full sample reported below. No evidence of differences existed between conditions in prior attitudes, intention to get vaccinated or confidence (all *p*s ≥ .084 in 2 × 2 × 2 ANOVAs).

**Figure 3:**
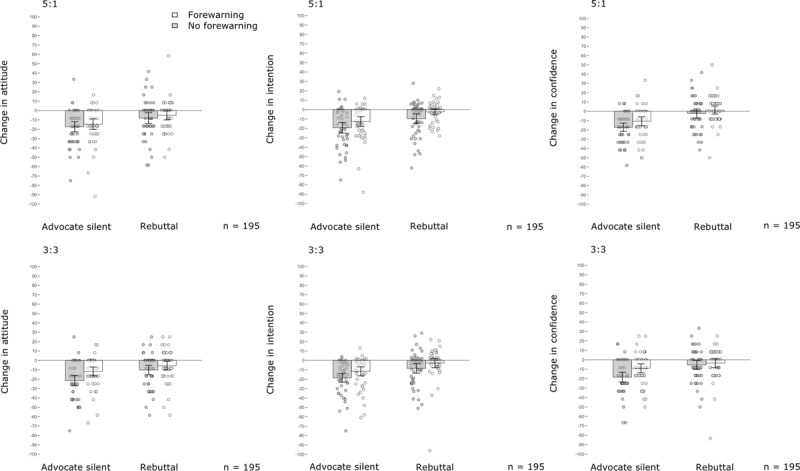
**Effects from outnumbering and forewarning on damage from science denialism in public discussions in Experiment 2.** The results reveal a significant mitigation in damage from denialism on all outcome measures when forewarning was used. The mitigating effect was not a function of whether the advocate uses a rebuttal or remains silent. Comparison of the lower (3:3) and upper (5:1) panels reveals no evidence that outnumbering mitigated the damage from denialism on any outcome measure. The y-axes represent mean changes in attitude (left graph), intention (centre graph) and confidence (right graph) in POMP values. Descriptive data are provided in Supplementary Tables 2–4. The x-axes represent experimental conditions. Error bars represent 95% CIs. Dots indicate changes in individual participants’ outcome measures.

Tables 2, 3, 4 and Figure 3 present the results of the 2 × 2 × 2 × 2 repeated-measures ANOVA models for individuals’ attitudes, confidence and intention. Replicating the results from Experiment 1, watching the public debate significantly damaged individuals’ attitudes towards vaccination, including intention to get vaccinated and confidence in vaccination. However, the rebuttal mitigated this damage on all outcome measures, again indicating successful manipulation.

**Table 4:** **Weight-of-evidence strategies’ effects on changes in confidence.** Significant effects are shown in boldface for the significance level of *p* < 0.05.

Confidence	Experiment 2 n = 390			Experiment 3 n = 396		
	
Effects	*F*	*p*	η^2^_*p*_	*F*	*p*	η^2^_*p*_

Time	**96.20**	**<.001**	**.201**	**61.59**	**<.001**	**.137**
Rebuttal × Time	**46.15**	**<.001**	**.108**	**70.75**	**<.001**	**.154**
Outnumbering × Time	1.35	.246	.004	0.81	.369	.002
Forewarning × Time	**10.44**	**.001**	**.027**	**8.76**	**.003**	**.022**
Rebuttal × Outnumbering × Time	1.72	.190	.004	0.03	.853	<.001
Rebuttal × Forewarning × Time	2.27	.132	.006	0.04	.949	<.001
Outnumbering × Forewarning × Time	0.04	.849	<.001	1.14	.287	.003
Rebuttal × Outnumbering × Forewarning × Time	0.49	.485	.001	2.21	.138	.006

Contrary to the findings in Experiment 1 and contradicting the rebuttal-as-moderator hypothesis, no evidence existed that a higher proportion of advocates present in the debate mitigated deniers’ influence on individuals’ attitude towards vaccination: *F*(1, 382) = 0.27, *p* = .605, η^2^*_p_* = .001; intention to get vaccinated: *F*(1, 382) = 0.15, *p* = .703, η^2^*_p_* < .001 or confidence: *F*(1, 382) = 1.35, *p* = .246, η^2^*_p_* = .004, not even when analysed as a function of whether or not a rebuttal was delivered (Tables 2, 3, 4). However, analyses revealed promising results on the forewarning hypothesis. A consistent mitigating effect was found when the audience was forewarned about false balance prior to the discussion compared with the control condition on all outcome measures, that is, attitude: *F*(1, 382) = 7.52, *p* = .006, η^2^*_p_* = .019; intention: *F*(1, 382) = 14.75, *p* < .001, η^2^*_p_* = .037; confidence: *F*(1, 382) = 10.44, *p* = .001, η^2^*_p_* = .027.

Confirmatory analyses were repeated with preregistered control variables (Supplementary Tables 5–6 and 8) and with a reduced sample containing only participants who provided correct answers to all three questions of the attention check (Supplementary Table 9–11), obtaining the same patterns of results.

Further exploration of the ANOVA models revealed no evidence that forewarning’s efficacy was a function of whether or not a rebuttal was delivered (Tables 2, 3, 4; Figure 3). We also found no evidence of a moderating effect from individuals’ issue involvement on the efficacy of any of the two weight-of-evidence strategies (Supplementary Table 7).

### Discussion

As in Experiment 1, we again found no evidence that outnumbering increases the audience’s resistance to science denialism. Thus, in the third experiment, we aimed to increase outnumbering’s persuasive power by adding another mechanism by which outnumbering potentially can work. Moreover, we aimed to replicate findings from Experiment 2 regarding the forewarning hypothesis.

## Experiment 3

In the previous experiments, only one denier and one advocate spoke during the discussion, regardless of the relative number of guests present during the debate. Thus, outnumbering was expected to work as a social cue. In this experiment, we tested another possible mechanism: outnumbering by delivering multiple rebuttal sources. With this change, we aimed to test whether inviting a greater number of science advocates compared with deniers is an effective weight-of-evidence strategy when all participants take part in the conversation. This additional effect can be expected only when the advocates do not remain silent. Thus, we preregistered the rebuttal-as-moderator hypothesis. Moreover, we preregistered the forewarning hypothesis, explored the rebuttal-as-moderator research question for forewarning and explored the involvement-as-moderator research question for both weight-of-evidence strategies. We preregistered the experiment at http://aspredicted.org/blind.php?x=wk8nc8.

### Method

The experimental setup was identical to Experiments 1 and 2, with deviations reported below.

#### Participants and design

A-priori power analyses using G*Power (Faul et al., 2009) revealed a minimum sample size of *N* = 368 to detect an interaction effect (minimum effect size of interest of *d* = 0.20) with a power of .80 in a mixed within-between ANOVA (eight groups, two measurements, α = .05). The effect size of the power analysis was chosen to detect conventionally small effect sizes (J. Cohen, 1977). Altogether, 369 undergraduate students from the University of Erfurt and RWTH Aachen University participated in this lab experiment in exchange for credit points (€4 or €5, depending on the laboratories’ different payment policies). Due to a technical error, 13 students could not enter demographic data. The study design was identical to that of Experiment 2. Again, we did not preregister any exclusion criteria, but stratified results based on individuals’ attention.

#### Materials and procedure

The number of advocates and deniers was equal to the number of speakers (Figure 1), that is, they served as multiple sources rather than a mere social cue. The arguments used in Experiments 1 and 2 were divided among all speakers. Thus, the total number of arguments remained constant across experiments. The forewarning used in this experiment was identical to the forewarning used in Experiment 2.

#### Measures

All measures were identical to Experiment 2 (Table 1, Supplementary Table 1).

### Results

Sixty-seven percent of the sample was female, with a mean age of 22.96 years (*SD*_age_ = 4.72) for all participants. In the end, 99.5% of the sample passed the content question, 82.3% correctly recalled the number of advocates and 85.1% correctly recalled the number of deniers who were present at the debate. Thus, we again repeated the primary analyses with a reduced sample and reported differences to the full sample. No evidence existed of differences between conditions in prior intentions to get vaccinated or prior confidence in vaccination, all *p*s ≥ .160 in 2 × 2 × 2 ANOVA. However, a difference did exist between conditions in individuals’ prior attitudes towards vaccination, as revealed through a significant three-way interaction, *F*(7, 388) = 4.08, *p* = .044, η^2^*_p_* = .010, in the 2 × 2 × 2 ANOVA (all other *p*s ≥ .198). Details of the interaction effect are reported in Supplementary Figure 1. To increase the findings’ robustness, the primary analysis of individuals’ attitudes was repeated using postvalues at T2, controlled for values at T1, rather than difference scores. Differences are reported below.

**Figure 4:**
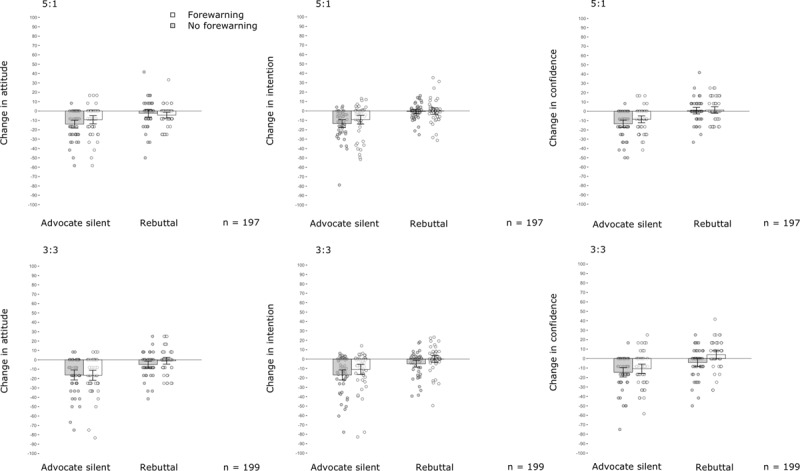
**Effects from outnumbering and forewarning on damage from science denialism in public discussions in Experiment 3.** Results reveal a significant mitigation of damage from denialism in individuals’ intention to get vaccinated and confidence in vaccination when forewarning was used. The mitigating effect was not a function of whether the advocate uses a rebuttal or remains silent. Comparison of the lower (3:3) and upper (5:1) panels reveals no evidence that outnumbering mitigated the damage from denialism on any outcome measure. The y-axes represent mean changes in attitude (left graph), intention (centre graph) and confidence (right graph) in POMP values. Descriptive data are provided in Supplementary Tables 2–4. The x-axes represent experimental conditions. Error bars represent 95% CIs. Dots indicate individual changes in individual participants’ outcome measures.

Tables 2, 3, 4 and Figure 4 present the 2 × 2 × 2 × 2 repeated-measures ANOVA models for individuals’ attitudes, confidence and intention. Again, attitudes, intention and confidence were damaged after watching the public discussion and the rebuttal mitigated this damage, confirming a successful manipulation.

Even with all advocates speaking, we again found no evidence for the outnumbering hypothesis, nor for the rebuttal-as-moderator hypothesis: A higher proportion of advocates did not mitigate the denier’s influence on individuals’ attitudes towards vaccination: *F*(1, 388) = 2.10, *p* = .148, η^2^*_p_* = .005; intention to get vaccinated: *F*(1, 388) = 2.44, *p* = .119, η^2^*_p_* = .006, or confidence: *F*(1, 388) = 0.81, *p* = .369, η^2^*_p_* = .002, not even when analysed as a function of rebuttal (Tables 2, 3, 4). Again, we found confirming evidence for the forewarning hypothesis, as damage from denialism was reduced in the audience that was forewarned about false balance compared with the control group, intention: *F*(1, 388) = 6.92, *p* = .009, η^2^*_p_* = .018; confidence: *F*(1, 388) = 8.76, *p* = .003, η^2^*_p_* = .022. This trend also was observed for individuals’ attitudes; however, the benefit from using forewarning remained not significant for this outcome, *F*(1, 388) = 1.11, *p* = .293, η^2^*_p_* = .003.

Confirmatory analyses were repeated with preregistered control variables (Supplementary Tables 5 and 6 and 8) and a sample adjusted for failing attention (Supplementary Tables 9–11). In addition, and due to differences in initial attitude values between conditions, we repeated the ANOVA for individuals’ attitudes using the attitude at T2 as a dependent variable controlling for attitude at T1, rather than difference scores (Supplementary Table 12). The patterns of results did not differ.

We found no evidence that forewarning’s efficacy was a function of whether a rebuttal was delivered (Tables 2, 3, 4) or a function of individuals’ issue involvement (Supplementary Table 7). We found individuals’ issue involvement to be a significant moderator of the efficacy of outnumbering on individuals’ attitudes towards vaccination. Less issue involvement led to greater damage from deniers when science advocates outnumbered deniers, while this damaging trend was reversed in the falsely balanced discussion (Supplementary Figure 2). This finding is difficult to interpret. In addition, we found no evidence of a moderation effect on individuals’ confidence in vaccination or intention to get vaccinated (Supplementary Table 7).

In the preregistration, we also planned to analyse whether the effects from forewarning and rebuttal were mediated by the denier’s perceived expertise. However, we accidentally did not ensure causal ordering because perceived expertise was measured after the dependent variables. We thus refrain from conducting this analysis.

### Discussion

Again, the forewarning mitigated the damage from vaccine denialism messages on the audience’s specific attitudinal beliefs, that is, vaccination safety and trust in institutions. Moreover, it mitigated the damage to the audience’s intention to vaccinate. Thus, forewarnings proved to be a helpful weight-of-evidence strategy against misinformation. As a limitation, and contrary to Experiment 2, we found no significant mitigating effect from forewarnings on the damage to the audience’s general attitudes towards vaccination. Despite our efforts to increase the persuasive strength of adding more advocates via the multiple-source mechanism, we again found no evidence that outnumbering mitigates science denialism damage from public discussions.

## General Discussion

The results from these three experiments provide some new insights into how editors and journalists can support the evidence-based voice of science when they invite science advocates and deniers to a public discussion. The results also showed that it is necessary to use such measures consciously, as in all three experiments, the science denier damaged study participants’ vaccination-related attitudes and intention, and reduced their confidence in vaccines’ safety and efficacy. While the damage can be mitigated through clever rebuttals from the advocate (replicating Schmid & Betsch, 2019), it cannot always be guaranteed that rebuttals will be delivered successfully – or at all (WHO, 2016).

In the light of the present findings, we expect that forewarnings about the false-balance effect should help reduce damage when screened prior to a falsely balanced discussion. The results from the present experiments reveal that such forewarnings are an effective weight-of-evidence strategy that can mitigate science denialism’s influence, independent of whether a rebuttal is delivered and independent of audience characteristics. The results are consistent with an increasing body of evidence showing that using prior information as a prebunking is an effective strategy against damage from misinformation (Cook et al., 2017; Roozenbeek & van der Linden, 2019). The use of prior information in the fight against misinformation has been shown to be effective in a variety of different contemporary issues (climate change: Cook et al., 2017; pandemics: Pennycook et al., in press). For example, Pennycook et al. (in press) discovered that motivating people to focus on the accuracy of headlines can reduce individuals’ willingness to share false statements about COVID-19. Pennycook et al. employ a simple accuracy reminder prior to misinformation, that is, they ‘ask people to assess the accuracy of a non-COVID-19-related headline’ (Pennycook et al., in press, p. 1). Similarly, the forewarning applied in this study simply explained false balance and mentioned the possibility of exposure to it in the subsequent public discussion. The forewarning did not specifically mention whether false balance was actually an issue in the subsequent discussion. Thus, it was up to the audience to identify whether the warning was applicable. This additional uncertainty might explain the rather small effect size of this weight-of-evidence strategy compared with previous findings on forewarnings’ impact (Quinn & Wood, 2003). On the positive side, it also means that even rather generic forewarning is helpful in protecting audiences against misinformation. Furthermore, such generic forewarning offers a specific economic advantage. It can be used for multiple shows, for example, on an online media platform, and does not need to be revised for every single public discussion that is broadcast. Therefore, future studies should test the forewarning effect’s duration and how specific or generic the warning may be in order to be effective.

In contrast to forewarnings’ efficacy as a weight-of-evidence strategy, we find no evidence that inviting more science advocates than deniers mitigates science denialism messages’ influence. The strategy of outnumbering science deniers had no success whatsoever, neither when silent advocates further served as a social cue representing the majority (Experiment 1–3), nor when lots of advocates served as multiple information sources (Experiment 3). Thus, the outnumbering strategy remained ineffective in the present experiments, even in audiences that, following dual-process theories, are likely to be persuaded by such peripheral cues.

The unexpected inefficacy of this weight-of-evidence strategy may be a result of the numeric relation between the majority and minority. Studies show that individuals are persuaded by a consensus when majorities become overwhelming (Lewandowsky et al., 2012). The numeric relation used in the present study (5:1) might fail to communicate such an overwhelming majority. However, Yousif, Aboody and Keil (2019) found that an even lower 4:1 distribution of positive vs. negative statements significantly influenced confidence in the majority’s position compared with a 1:1 distribution of statements. Thus, the stimulus material used in this study seems adequate for detecting an effect from the distribution of speakers if such an effect exists in the context of public discussions.

Another potential concern with the materials used in this study is the dependence on multiple sources. One of the very first studies about the multiple-source effect by Harkins and Petty (1987) found that the advantage of having multiple sources is a function of the sources’ independence, that is, multiple sources that can be attributed to the same origin are not more persuasive than a single source, while independent sources lead to the majority’s expected persuasive advantage (Harkins & Petty, 1987). In the present study, science advocates were described as employees of the same agency, so they may have lost their persuasive advantage due to their shared employer. However, Yousif, Aboody and Keil (2019) found that information shared by multiple sources was more persuasive than information from a single source, even when statements from multiple sources depended on the same primary source. Thus, in light of current findings, we have no evidence to believe that a mere weighted distribution of speakers in a public discussion could mitigate science denialism’s influence.

### Limitations

This study has several limitations. First, consistent with other survey experiments of this kind (Nyhan et al. 2019, Barrera et al. 2020), the findings on the efficacy of the forewarning may be influenced by a demand effect. It may seem difficult to generally separate a forewarning effect from a demand effect, as individuals are demanded not to use a particular type of information. This has implications for the internal and external validity. Recent research indicates that demand effects are rather rare in survey experiments; thus, we assume that the threat of the internal validity is rather low (Mummolo and Peterson, 2019). As forewarnings are usually implemented by health and media authorities and the experimenter may be seen as an authority, the experimental setting mirrors real-world settings, which should increase the external validity (Zizzo, 2010). The efficacy of forewarnings may be a function of the participants’ perceived authority of the user of these strategies, which remains subject to further investigation. Second, we used a student sample which was both a strength and a limitation. On the one hand, selecting student samples was a strong test for the hypothesis that messages of science denial damage the intention to get vaccinated and damage the attitude towards vaccination as student samples are considered more educated than average and therefore more resistant to misinformation (Walter & Murphy, 2018). On the other hand, the selection of student samples limits the generalisability of results. Previous online experiments with heterogeneous samples in Germany and the U.S. report similar patterns of results (Schmid & Betsch, 2019) regarding science deniers’ influence and the efficacy of rebuttal messages. However, the outlined weight-of-evidence strategies in this study have not been tested with other samples, that is, the findings may vary with different audiences. For example, forewarning about the false-balance effect might be less effective among less-educated audiences compared with highly educated undergraduates. Future studies will address this question. A third limitation is the presented scenario’s fictitious nature. The choice to use these fictitious scenarios in studies about vaccination decisions in this study and previous publications (Schmid & Betsch, 2019) is primarily based on ethical considerations. However, this choice may reduce the presented findings’ external validity.

## Conclusion

Given the results, editors, journalists and other mass media outlets should invest some effort in providing forewarnings as an effective weight-of-evidence strategy. No evidence was obtained that the efficacy of forewarnings depends on advocates’ delivery of specific rebuttals in the discussion or the audience’s issue involvement. Therefore, we suggest that warning audiences about false-balance reporting prior to debates can serve as a theory-driven, economic and effective weight-of-evidence strategy to support advocates for science in public discussions about scientific topics. To increase the generalisability of the findings, future studies will benefit from analysing the efficacy of weight-of-evidence strategies as a function of varying sample characteristics and participants’ perceived authority of the user of these strategies.

## Data Accessibility Statement

The materials, data and syntax supporting the findings of this study are archived in the Open Science Framework’s public database and are available to anyone: https://doi.org/10.17605/OSF.IO/SEFQU.

## Additonal File

The additonal file for this article can be found as follows:

10.5334/joc.125.s1 Supplementary Information. Supplementary Method and Supplementary Results.
