# Supplementary material for: Weight-of-Evidence Strategies to Mitigate the Influence of Messages of Science Denialism in Public Discussions
Source: J Cogn. 2020 Oct 1;3(1):36. doi: 10.5334/joc.125 (PMC7528676; doi:10.5334/joc.125)
Supplement: Supplementary Information. — Supplementary Method and Supplementary Results. [file joc-3-1-125-s1.pdf]

Supplementary Information

Weight-of-Evidence Strategies to Mitigate the Influence of Messages of Science Denialism in Public Discussions

Supplementary Method

Descriptive data for participants

Supplementary Material 1: Messages delivered by deniers and advocates for all conditions and experiments

Supplementary Material 2: Forewarning received by control group in Experiments 2 and 3

Supplementary Table 1. Overview of additional measures

Supplementary Results

Supplementary Table 2. Descriptive data for changes in attitude

Supplementary Table 3. Descriptive data for changes in intention

Supplementary Table 4. Descriptive data for changes in confidence

Supplementary Table 5. Effects from rebuttal and weight-of-evidence strategies on changes in intention after controlling for preregistered covariates' effects

Supplementary Table 6. Effects from rebuttal and weight-of-evidence strategies on attitude changes after controlling for preregistered covariates' effects

Supplementary Table 7. The efficacy of inviting more advocates as a function of individuals' issue involvement

Supplementary Table 8. The effects from rebuttal and weight-of-evidence strategies on changes in confidence after controlling for preregistered covariates' effects

Supplementary Table 9. Effects from rebuttal and weight-of-evidence strategies on changes in intention with a sample containing only those participants who recalled the correct information

Supplementary Table 10. Effects from rebuttal and weight-of-evidence strategies on attitude changes with a sample containing only those participants who recalled the correct information

Supplementary Table 11. Effects from rebuttal and weight-of-evidence strategies on changes in confidence with a sample containing only those participants who recalled the correct information

Supplementary Table 12. Effects from rebuttal and weight-of-evidence strategies on post-attitude values controlled for pre-attitude values

Supplementary Figure 1. Evidence from a biased distribution of pre-values in Experiment 3.

Supplementary Figure 2. Outnumbering increases the damage from denialism with increasing issue involvement from the audience.

Supplementary References

Descriptive data for participants

Experiment 1:

Participants in Experiment 1 indicated a positive attitude towards vaccination against the fictional disease dysomeria ($M_{prior\_attitude}$ = 77.48, $SD_{prior\_attitude}$ = 18.35) and a rather high willingness to get vaccinated ($M_{prior\_intention}$ = 72.60, $SD_{prior\_intention}$ = 24.12) before watching the public discussion. On average, they reached 49.6% ($M_{knowledge}$ = 49.62, $SD_{knowledge}$ = 24.52) of the maximum possible score for knowledge about vaccination in general. Participants assigned low relevance to TV as an information source on vaccination ($M_{relevance\_TV}$ = 11.05, $SD_{relevance\_TV}$ = 10.70) and reported rather low involvement in vaccination in general ($M_{involvement}$ = 36.37, $SD_{involvement}$ = 17.12).

Experiment 2:

Similar to Experiment 1, participants indicated a positive attitude towards vaccination against dysomeria ($M_{prior\_attitude}$ = 70.98, $SD_{prior\_attitude}$ = 20.96), a rather high willingness to get vaccinated ($M_{prior\_intention}$ = 68.34, $SD_{prior\_intention}$ = 23.71) and moderate confidence in vaccination against dysomeria ($M_{prior\_confidence}$ = 58.35, $SD_{prior\_confidence}$ = 19.60) before watching the public discussion. On average, they reached 45.6% ($M_{knowledge}$ = 45.64, $SD_{knowledge}$ = 23.55) of the maximum possible score for knowledge about vaccination in general. Participants assigned low relevance to TV as an information source on vaccination ($M_{relevance\_TV}$ = 9.73, $SD_{relevance\_TV}$ = 9.88) and reported rather low involvement in vaccination in general ($M_{involvement}$ = 36.37, $SD_{involvement}$ = 17.12).

Experiment 3:

Similar to the previous experiments, participants indicated a positive attitude towards vaccination against dysomeria ($M_{prior\_attitude}$ = 70.98, $SD_{prior\_attitude}$ = 20.96), a high willingness

to get vaccinated ($M_{prior\_intention}$ = 68.34, $SD_{prior\_intention}$ = 23.71) and rather high confidence in vaccination against dysomeria ($M_{prior\_confidence}$ = 70.98, $SD_{prior\_confidence}$ = 20.96) before watching the public discussion. On average, they reached 55.1% ($M_{knowledge}$ = 55.10, $SD_{knowledge}$ = 24.37) of the maximum possible score for knowledge about vaccination in general. Again, participants assigned low relevance to TV as an information source on vaccination ($M_{relevance\_TV}$ = 11.01, $SD_{relevance\_TV}$ = 10.65) and reported rather low involvement in vaccination in general ($M_{involvement}$ = 32.70, $SD_{involvement}$ = 15.53).

Supplementary Material 1. Messages delivered by deniers and advocates for all conditions and experiments. Note: original materials were in German.

Experiments 1 and 2

Rebuttal: Advocate silent
Outnumbering: 5:1

1. Video

Florian Hantzsch (Interviewer): ‘Welcome, ladies and gentlemen. Today, we are talking about the vaccine against the viral disease dysomeria. I am delighted to welcome my six guests: Mr. Stefan Müller from the Neustaedter Vaccine-Sceptics and Mrs. Natalia Holderman, Mr. Domenik Rehde, Mr. Martin Schober, Mr. Peter Witting and Mr. Jürgen Schmidt from the Health Office in Neustadt.
I would like to start right away with my first question:
Mr. Stefan Müller from the Neustaedter Vaccine-Sceptics, how safe is the vaccine against dysomeria?’

Stefan Müller (Science Denier 1): ‘The lack of safety is an important issue with the dysomeria vaccine. The side effects and risks of the vaccine are incalculable. As a patient, you do not know how the body reacts to the vaccine before administration. Even if you feel healthy immediately after the shot, harmful substances may have entered your body. Doctors cannot guarantee in advance that there will not be any complications. In my opinion, you cannot expect any fellow citizens to vaccinate as long as the vaccine is not 100% safe. Surely, it is not too much to ask that a product injected into a healthy human body be 100% safe.’

2. Video

Florian Hantzsch (Interviewer): ‘Vaccines protect against diseases; however, pharmaceutical companies also make money from producing vaccines. A question arises: Who actually benefits from vaccination, Mr. Müller?’

Stefan Müller (Science Denier 1): ‘At the end of the day, it is not about the health of the individual citizen. It is about the financial interests of large companies and government institutions. The pharmaceutical industry earns a huge annual profit from the sale of the vaccine against dysomeria. The government can multiply the profit tremendously with official vaccination recommendations. If we put two and two together, then anyone can see this perfidious collaboration between the responsible parties. In the end, all those who have something to say in this system are connected in a way, and only the ordinary citizen is left out – and is expected to do one thing: Stay silent and keep on vaccinating.’

Rebuttal: Advocate silent
Outnumbering: 3:3

1. Video

Florian Hantzsch (Interviewer): ‘Welcome, ladies and gentlemen. Today, we are talking about the vaccine against the viral disease dysomeria. I am delighted to welcome my six guests: Mr. Domenik Rehde, Mrs. Natalia Holderman and Mr. Stefan Müller from the Neustaedter Vaccine-Sceptics and also Mr. Martin Schober, Mr. Peter Witting and Mr. Jürgen Schmidt from the Health Office in Neustadt. I would like to start right away with my first question:
Mr. Stefan Müller from the Neustaedter Vaccine-Sceptics, how safe is the vaccine against dysomeria?’

Stefan Müller (Science Denier 1): 'The lack of safety is an important issue with the dysomeria vaccine. The side effects and risks from the vaccine are incalculable. As a patient, you do not know how the body reacts to the vaccine before administration. Even if you feel healthy immediately after the shot, harmful substances may have entered your body. Doctors cannot guarantee in advance that there will not be any complications. In my opinion, you cannot expect any fellow citizens to vaccinate as long as the vaccine is not 100% safe. Surely, it is not too much to ask that a product injected into a healthy human body be 100% safe.'

2. Video

Florian Hantzsch (Interviewer): 'Vaccines protect against diseases; however, pharmaceutical companies also make money from producing vaccines. A question arises: Who actually benefits from vaccination, Mr. Müller?'

Stefan Müller (Science Denier 1): 'At the end of the day, it is not about the health of the individual citizen. It is about the financial interests of large companies and government institutions. The pharmaceutical industry earns a huge annual profit with the sale of the vaccine against dysomeria. The government can multiply the profit tremendously with official vaccination recommendations. If we put two and two together, then anyone can see this perfidious collaboration between the responsible parties. In the end, all those who have something to say in this system are connected in a way, and only the ordinary citizen is left out – and is expected to do one thing: Stay silent and keep on vaccinating.'

Rebuttal: Yes
Outnumbering: 5:1

1. Video

Florian Hantzsch (Interviewer): 'Welcome, ladies and gentlemen. Today, we are talking about the vaccine against the viral disease dysomeria. I am delighted to welcome my six guests: Mr. Stefan Müller from the Neustaedter Vaccine-Sceptics and also Mrs. Natalia Holderman, Mr. Domenik Rehde, Mr. Martin Schober, Mr. Peter Witting and Mr. Jürgen Schmidt from the Health Office in Neustadt. I would like to start right away with my first question:
Mr. Stefan Müller from the Neustaedter Vaccine-Sceptics, how safe is the vaccine against dysomeria?'

Stefan Müller (Science Denier 1): 'The lack of safety is an important issue with the dysomeria vaccine. The side effects and risks of the vaccine are incalculable. As a patient, you do not know how the body reacts to the vaccine before administration. Even if you feel healthy immediately after the shot, harmful substances may have entered your body. Doctors cannot guarantee in advance that there will not be any complications. In my opinion, you cannot expect any fellow citizen to vaccinate as long as the vaccine is not 100% safe. Surely, it is not too much to ask that a product injected into a healthy human be 100% safe.'

Florian Hantzsch (Interviewer): 'Thank you, Mr. Müller. Mr. Jürgen Schmidt from the Health Office in Neustadt, how do you respond to that?'

Jürgen Schmidt (Science Advocate 1): 'Mr. Müller demands 100% safety from the vaccine against dysomeria. In science, this argument is called *impossible expectation*. It is an impossible expectation because science can never guarantee 100% safety for any medical product, neither for aspirin nor for heart surgery. Any treatment poses a residual risk of complications for patients either during or after treatment. The scientific evidence is clear: The vaccine against dysomeria is a safe way to avoid the disease. The risk of dysomeria by far exceeds the risk from vaccination. This is why we, the Health Office in Neustadt, recommend the vaccination against the DS virus for citizens of all ages. And please let me add the following regarding the safety of the vaccine: We follow a very strict protocol to ensure the high quality of vaccines in the Federal States. This also is demonstrated by the fact that every batch of the vaccine against dysomeria constantly is monitored and independently screened by official control laboratories.'

2. Video

Florian Hantzsch (Interviewer): 'Vaccines protect against diseases; however, pharmaceutical companies also make money from producing vaccines. A question arises: Who actually benefits from vaccination, Mr. Müller?'

Stefan Müller (Science Denier 1): 'At the end of the day, it is not about the health of the individual citizen. It is about the financial interests of large companies and government institutions. The pharmaceutical industry earns a huge annual profit from the sale of the vaccine against dysomeria. The government can multiply the profit tremendously with official vaccination recommendations. If we put two and two together, then anyone can see this perfidious collaboration between the responsible parties. In the end, all those who have something to say in this system are connected in a way, and only the ordinary citizen is left out – and is expected to do one thing: Stay silent and keep on vaccinating.'

Florian Hantzsch (Interviewer): 'Thank you, Mr. Müller. Mr. Jürgen Schmidt from the Health Office in Neustadt, how do you respond to that?'

Jürgen Schmidt (Science Advocate 1): 'Mr. Müller suspects a secret conspiracy behind the distribution of the vaccine against dysomeria. This perspective completely ignores that a large proportion of the research that demonstrates the benefits of vaccination for society and each individual is conducted by independent scientists all over the world. In addition, such conspiratorial claims discredit the prosocial motives of all our healthcare system's employees. Let's stay with the facts: In

regions where the vaccine against dysomeria is used, people live a healthier life. This has been demonstrated several times. The major goal of governmental health institutions like our office is to maintain and improve the health of every single citizen in the country. I very much regret that Mr. Müller has lost trust in our institution and our effort. The Standing Committee on Vaccination, STIKO, which is responsible for vaccination recommendations in the Federal States, is composed of independent experts who are appointed for a period of three years. The members are an independent advisory group, and the meetings and protocols of the STIKO, as well as possible conflicts of interest among members, are open to the public and available via webcast. Whatever Mr. Müller is suggesting here, the fact is: The vaccine improves the health standard of all individuals, and that is why we recommend it for citizens of all ages.'

Rebuttal: Yes
Outnumbering: 3:3

1. Video

Florian Hantzsch (Interviewer): 'Welcome, ladies and gentlemen. Today, we are talking about the vaccine against the viral disease dysomeria.
I am delighted to welcome my six guests: Mr. Domenik Rehde, Mrs. Natalia Holderman and Mr. Stefan Müller from the Neustaedter Vaccine-Sceptics and also Mr. Martin Schober, Mr. Peter Witting and Mr. Jürgen Schmidt from the Health Office in Neustadt.
I would like to start right away with my first question:
Mr. Stefan Müller from the Neustaedter Vaccine-Sceptics, how safe is the vaccine against dysomeria?'

Stefan Müller (Science Denier 1): 'The lack of safety is an important issue with the dysomeria vaccine. The side effects and risks of the vaccine are incalculable. As a patient, you do not know how the body reacts to the vaccine before administration. Even if you feel healthy immediately after the shot, harmful substances may have entered your body. Doctors cannot guarantee in advance that there will not be any complications. In my opinion, you cannot expect any fellow citizen to vaccinate as long as the vaccine is not 100% safe. Surely, it is not too much to ask that a product injected into a healthy human body be 100% safe.'

Florian Hantzsch (Interviewer): 'Thank you, Mr. Müller. Mr. Jürgen Schmidt from the Health Office in Neustadt, how do you respond to that?'

Jürgen Schmidt (Science Advocate 1): 'Mr. Müller demands 100% safety from the vaccine against dysomeria. In science, this argument is called *impossible expectation*. It is an impossible expectation because science can never guarantee 100% safety for any medical product, neither for aspirin nor for heart surgery. Any treatment poses a residual risk of complications for patients either during or after treatment. The scientific evidence is clear: The vaccine against dysomeria is a safe way to avoid the disease. The risk of dysomeria by far exceeds the risk from vaccination. This is why we, the Health Office in Neustadt, recommend the vaccination against the DS virus for citizens of all ages. And please let me add the following regarding the safety of the vaccine: We follow a very strict protocol to ensure the high quality of vaccines in the Federal States. This is also demonstrated by the fact that every batch of the vaccine against dysomeria is monitored constantly and independently screened by official control laboratories.'

2. Video

Florian Hantzsch (Interviewer): 'Vaccines protect against diseases; however, pharmaceutical companies also make money from producing vaccines. A question arises: Who actually benefits from vaccination, Mr. Müller?'

Stefan Müller (Science Denier 1): 'At the end of the day, it is not about the health of the individual citizen. It is about the financial interests of large companies and government institutions. The pharmaceutical industry earns a huge annual profit with the sale of the vaccine against dysomeria. The government can multiply the profit tremendously with official vaccination recommendations. If we put two and two together, then anyone can see this perfidious collaboration between the responsible parties. In the end, all those who have something to say in this system are connected in a way, and only the ordinary citizen is left out – and is expected to do one thing: Stay silent and keep on vaccinating.'

Florian Hantzsch (Interviewer): 'Thank you, Mr. Müller. Mr. Jürgen Schmidt from the Health Office in Neustadt, how do you respond to that?'

Jürgen Schmidt (Science Advocate 1): 'Mr. Müller suspects a secret conspiracy behind the distribution of the vaccine against dysomeria. This perspective completely ignores that a large proportion of the research that demonstrates the benefits of vaccination for society and each individual is conducted by independent scientists all over the world. In addition, such conspiratorial claims discredit the prosocial motives of all our healthcare system's employees. Let's stay with the facts: In regions where the vaccine against dysomeria is used, people live a healthier life. This has been demonstrated several times. The major goal of governmental health institutions like our office is to maintain and improve the health of every single citizen in the country. I very much regret that Mr. Müller has lost trust in our institution and our effort. The Standing Committee on Vaccination STIKO, which is responsible for vaccination recommendations in the Federal States, is composed of independent experts who are appointed for a period of three years. The members are an independent advisory group, and the meetings and protocols of the STIKO, as well as possible conflicts of interest among the members, are open to the public and available via webcast. Whatever Mr. Müller is suggesting here, the fact is: The vaccine improves the health standards of all individuals, and that is why we recommend it for citizens of all ages.'

Experiment 3

Rebuttal: Advocate silent
Outnumbering: 5:1

1. Video

Florian Hantzsch (Interviewer): 'Welcome, ladies and gentlemen. Today, we are talking about the vaccine against the viral disease dysomeria. I am delighted to welcome my six guests: Mr. Stefan Müller from the Neustaedter Vaccine-Sceptics and also Mr. Leon Holderman, Mr. Domenik Rehde, Mr. Martin Schober, Mr. Peter Witting and Mr. Jürgen Schmidt from the Health Office in Neustadt.
I would like to start right away with my first question:
Mr. Stefan Müller from the Neustaedter Vaccine-Sceptics, how safe is the vaccine against dysomeria?'

Stefan Müller (Science Denier 1): 'The lack of safety is an important issue with the dysomeria vaccine. The side effects and risks of the vaccine are incalculable. As a patient, you do not know how the body reacts to the vaccine before administration. Even if you feel healthy immediately after the shot, harmful substances may have entered your body. Doctors cannot guarantee in advance that there will not be any complications. In my opinion, you cannot expect any fellow citizen to vaccinate as long as the vaccine is not 100% safe. Surely, it is not too much to ask that a product injected into a healthy human body be 100% safe.'

2. Video

Florian Hantzsch (Interviewer): 'Vaccines protect against diseases; however, pharmaceutical companies also make money from producing vaccines. A question arises: Who actually benefits from vaccination, Mr. Müller?'

Stefan Müller (Science Denier 1): 'At the end of the day, it is not about the health of the individual citizen. It is about the financial interests of large companies and government institutions. The pharmaceutical industry earns a huge annual profit with the sale of the vaccine against dysomeria. The government can multiply the profit tremendously with official vaccination recommendations. If we put two and two together, then anyone can see this perfidious collaboration between the responsible parties. In the end, all those who have something to say in this system are connected in a way, and only the ordinary citizen is left out – and is expected to do one thing: Stay silent and keep on vaccinating.'

Rebuttal: Advocate silent
Outnumbering: 3:3

1. Video

Florian Hantzsch (Interviewer): 'Welcome, ladies and gentlemen. Today, we are talking about the vaccine against the viral disease dysomeria. I am delighted to welcome my six guests: Mr. Domenik Rehde, Mr. Leon Holderman and Mr. Stefan Müller from the Neustaedter Vaccine-Sceptics and also Mr. Martin Schober, Mr. Peter Witting and Mr. Jürgen Schmidt from the Health Office in Neustadt.
I would like to start right away with my first question:
Mr. Stefan Müller from the Neustaedter Vaccine-Sceptics, how safe is the vaccine against dysomeria?'

Stefan Müller (Science Denier 1): 'The lack of safety is an important issue with the dysomeria vaccine. The side effects and risks of the vaccine are incalculable. As a patient, you do not know how the body reacts to the vaccine before administration. Even if you feel healthy immediately after the shot, harmful substances may have entered your body. Doctors cannot guarantee in advance that there will not be any complications. In my opinion, you cannot expect any fellow citizen to vaccinate as long as the vaccine is not 100% safe. Surely, it is not too much to ask that a product injected into a healthy human body be 100% safe.'

2. Video

Florian Hantzsch (Interviewer): 'Vaccines protect against diseases; however, pharmaceutical companies also make money from producing vaccines. A question arises: Who actually benefits from vaccination, Mr. Rehde?'

Domenik Rehde (Science Denier 2): 'At the end of the day, it is not about the health of the individual citizen. It is about the financial interests of large companies and government institutions. The pharmaceutical industry earns a huge annual profit from the sale of the vaccine against dysomeria. The government can multiply the profit tremendously with official vaccination recommendations.'

Florian Hantzsch (Interviewer): 'Thank you, Mr. Rehde. Mr. Holderman, what do you think?'

Leon Holderman (Science Denier 3): 'If we put two and two together, then anyone can see this perfidious collaboration between the responsible parties. In the end, all those who have something to say in this system are connected in a way, and only the ordinary citizen is left out – and is expected to do one thing: Stay silent and keep on vaccinating.'

Rebuttal: Yes
Outnumbering: 5:1

1. Video

Florian Hantzsch (Interviewer): 'Welcome, ladies and gentlemen. Today, we are talking about the vaccine against the viral disease dysomeria. I am delighted to welcome my six guests: Mr. Stefan Müller from the Neustaedter Vaccine-Sceptics and also Mr. Leon Holderman, Mr. Domenik Rehde, Mr. Martin Schober, Mr. Peter Witting and Mr. Jürgen Schmidt from the Health Office in Neustadt.
I would like to start right away with my first question:
Mr. Stefan Müller from the Neustaedter Vaccine-Sceptics, how safe is the vaccine against dysomeria?'

Stefan Müller (Science Denier 1): 'The lack of safety is an important issue with the dysomeria vaccine. The side effects and risks of the vaccine are incalculable. As a patient, you do not know how the body reacts to the vaccine before administration. Even if you feel healthy immediately after the shot, harmful substances may have entered your body. Doctors cannot guarantee in advance that there will not be any complications. In my opinion, you cannot expect any fellow citizen to vaccinate as long as the vaccine is not 100% safe. Surely, it is not too much to ask that a product injected into a healthy human body be 100% safe.'

Florian Hantzsch (Interviewer): 'Thank you, Mr. Müller. Mr. Jürgen Schmidt from the Health Office in Neustadt, how do you respond to that?'

Jürgen Schmidt (Science Advocate 1): 'Mr. Müller demands 100% safety from the vaccine against dysomeria. In science, this argument is called *impossible expectation.* It is an impossible expectation because science can never guarantee 100% safety for any medical product, neither for aspirin nor for heart surgery. Any treatment poses a residual risk of complications for patients either during or after treatment.'

Florian Hantzsch (Interviewer): 'Thank you, Mr. Schmidt. Mr. Holderman, what do you think?'

Leon Holderman (Science Advocate 2): 'The scientific evidence is clear; the vaccine against dysomeria is a safe way to avoid the disease. The risk of dysomeria by far exceeds the risk from vaccination. That is why we at the Health Office in Neustadt recommend the vaccination against the DS virus for citizens of all ages. And please let me add the following regarding the safety of the vaccine: We follow a very strict protocol to ensure the high quality of vaccines in the Federal States. This is also demonstrated by the fact that every batch of the vaccine against dysomeria is constantly monitored and independently screened by official control laboratories.'

2. Video

Florian Hantzsch (Interviewer): 'Vaccines protect against diseases; however, pharmaceutical companies also make money from producing vaccines. A question arises: Who actually benefits from vaccination, Mr. Müller?'

Stefan Müller (Science Denier 1): 'At the end of the day, it is not about the health of the individual citizen. It is about the financial interests of large companies and government institutions. The pharmaceutical industry earns a huge annual profit with the sale of the vaccine against dysomeria. The government can multiply the profit tremendously with official vaccination recommendations. If we put two and two together, then anyone can see this perfidious collaboration between the responsible parties. In the end, all those who have something to say in this system are connected in a way, and only the ordinary citizen is left out – and is expected to do one thing: Stay silent and keep on vaccinating.'

Florian Hantzsch (Interviewer): 'Thank you, Mr. Müller. Mr. Martin Schober from the Health Office in Neustadt, how do you respond to that?'

Martin Schober (Science Advocate 3): 'In science, this argument is called *secret conspiracy*. This perspective completely ignores that a large proportion of the research that demonstrates the benefits of vaccination for society and each individual is conducted by independent scientists all over the world. In addition, such conspiratorial claims discredit the prosocial motives of all our healthcare system's employees.'

Florian Hantzsch (Interviewer): 'Thank you, Mr. Schober. Mr. Witting, what do you think?'

Peter Witting (Science Advocate 4): 'Let's stick with the facts: In regions where the vaccine against dysomeria is used, people live a healthier life. That has been demonstrated several times. The major goal of governmental health institutions like our office is to maintain and improve the health of every single citizen in the country.'

Florian Hantzsch (Interviewer): 'Thank you, Mr. Witting. Mr. Rehde, what do you think?'

Domenik Rehde (Science Advocate 5): 'The Standing Committee on Vaccination STIKO, which is responsible for vaccination recommendations in the Federal States, is composed of independent experts who are appointed for a period of three years. The members are an independent advisory group, and the meetings and protocols of the STIKO, as well as possible conflicts of interest among the members, are open to the public and available via webcast. The fact is: The vaccine improves the health standard of all individuals.'

Rebuttal: Yes
Outnumbering: 3:3

1. Video

Florian Hantzsch (Interviewer): 'Welcome, ladies and gentlemen. Today, we are talking about the vaccine against the viral disease dysomeria. I am delighted to welcome my six guests: Mr. Domenik Rehde, Mr. Leon Holderman and Mr. Stefan Müller from the Neustaedter Vaccine-Sceptics and also Mr. Martin Schober, Mr. Peter Witting and Mr. Jürgen Schmidt from the Health Office in Neustadt.
I would like to start right away with my first question:
Mr. Stefan Müller from the Neustaedter Vaccine-Sceptics, how safe is the vaccine against dysomeria?'

Stefan Müller (Science Denier 1): 'The lack of safety is an important issue with the dysomeria vaccine. The side effects and risks of the vaccine are incalculable. As a patient, you do not know how the body reacts to the vaccine before administration. Even if you feel healthy immediately after the shot, harmful substances may have entered your body. Doctors cannot guarantee in advance that there will not be any complications. In my opinion, you cannot expect any fellow citizen to vaccinate as long as the vaccine is not 100% safe. Surely, it is not too much to ask that a product injected into a healthy human body be 100% safe.'

Florian Hantzsch (Interviewer): 'Thank you, Mr. Müller. Mr. Jürgen Schmidt from the Health Office in Neustadt, how do you respond to that?'

Jürgen Schmidt (Science Advocate 1): 'Mr. Müller demands 100% safety from the vaccine against dysomeria. In science, this argument is called *impossible expectation*. It is an impossible expectation because science can never guarantee 100% safety for any medical product, neither for aspirin nor for heart surgery. Any treatment poses a residual risk of complications for patients either during or after treatment. The scientific evidence is clear: The vaccine against dysomeria is a safe way to avoid the disease. The risk of dysomeria by far exceeds the risk from vaccination. That is why we at the Health Office in Neustadt recommend vaccination against the DS virus for citizens of all ages. And please let me add the following regarding the safety of the vaccine: We follow a very strict protocol to ensure the high quality of vaccines in the Federal States. This is also demonstrated by the fact that every batch of the vaccine against dysomeria is constantly monitored and independently screened by official control laboratories.'

2. Video

Florian Hantzsch (Interviewer): 'Vaccines protect against diseases; however, pharmaceutical companies also make money from producing vaccines. A question arises: Who actually benefits from vaccination, Mr. Rehde?'

Domenik Rehde (Science Denier 2): 'At the end of the day, it is not about the health of the individual citizen. It is about the financial interests of large companies and government institutions. The pharmaceutical industry earns a huge annual profit with the sale of the vaccine against dysomeria. The government can multiply the profit tremendously with official vaccination recommendations.'

Florian Hantzsch (Interviewer): 'Thank you, Mr. Rehde. Mr. Holderman, what do you think?'

Leon Holderman (Science Denier 3): 'If we put two and two together, then anyone can see this perfidious collaboration between the responsible parties. In the end, all those who have something to say in this system are connected in a way, and only the ordinary citizen is left out – and is expected to do one thing: Stay silent and keep on vaccinating.'

Florian Hantzsch (Interviewer): 'Thank you, Mr. Holderman. Mr. Martin Schober from the Health Office in Neustadt, how do you respond to that?'

Martin Schober (Science Advocate 2): 'In science, this argument is called *secret conspiracy*. This perspective completely ignores that a large proportion of the research that demonstrates the benefits of vaccination for society and each individual is conducted by independent scientists all over the world. In addition, such conspiratorial claims discredit the pro-social motives of all our healthcare system's employees. Let's stick with the facts: In regions where the vaccine against dysomeria is used, people live a healthier life. That has been demonstrated several times.'

Florian Hantzsch (Interviewer): 'Thank you, Mr. Schober. Mr. Witting, what do you think?'

Peter Witting (Science Advocate 3): 'The major goal of governmental health institutions like our office is to maintain and improve the health of every single citizen in the country. The Standing Committee on Vaccination STIKO, which is responsible for vaccination recommendations in the Federal States, is composed of independent experts who are appointed for a period of three years. The members are an independent advisory group, and the meetings and protocols of the STIKO, as well as possible conflicts of interest among the members, are open to the public and available via webcast. The fact is: The vaccine improves the health standard of all individuals.'

Supplementary Material 2. Forewarning received by control group in Experiments 2 and 3. Note: original materials were in German.

Note!

Data protection is very important to us; therefore, we take protection of your data very seriously. We always want you to feel safe when using our Internet services and to know exactly which data are stored and used. We follow the principles of data avoidance and data economy. The basis for this is the law applicable in Germany in the form of the Federal Data Protection Act and the EU's Basic Data Protection Regulation.

All access to our websites and all file retrievals are recorded for statistical and security purposes. In addition, storage of accesses serves to guarantee system stability. To determine this data, our sender, as well as other community facilities, use so-called pixel-code data, which are collected and stored in anonymous form for optimisation and study purposes. These measurements were developed for data protection. Your identity is always protected. You will not receive any advertising via the system. We make every effort to protect your personal data from unauthorised access by means of organisational measures. Please note that data security on the Internet cannot be guaranteed when communicating via e-mail and that we recommend sending confidential information by post.

Enjoy the show.

Construct	Included in	Scale type and reliability*	wording	Source of items
**Moderator variable**				
issue involvement	Experiment 1–3	mean score of 7-point semantic differentials ($\alpha1$ = .87; $\alpha2$ = .83; $\alpha3$ = .84)	For me the subject vaccination is… (unimportant – important, relevant – irrelevant, essential – nonessential, fascinating – mundane, insignificant – significant, appealing – unappealing, boring – interesting)	Zaichkowsky (1985)
**Control variables**				
knowledge about vaccination	Experiment 1–3	mean score of correct/incorrect answers	Example item: Diseases like autism, multiple sclerosis, and diabetes might be triggered through vaccinations. (1 = yes, 2 = no, 3 = I do not know)	Zingg & Siegrist (2012)
5C – psychological antecedents of vaccination	Experiment 1–3	7-point rating scale	Please evaluate to what extent you disagree or agree with the following statements.<br><br>(Confidence) I am completely confident that vaccines are safe.<br>(Collective responsibility) When everyone is vaccinated, I don't have to get vaccinated, too.<br>(Constrains) Everyday stress prevents me from getting vaccinated.<br>(Complacency) Vaccination is unnecessary because vaccine-preventable diseases are not common anymore.<br>(Calculation) When I think about getting vaccinated, I weigh benefits and risks to make the best decision possible.<br>(1 = I strongly disagree, 5 = I strongly agree)	Betsch et al. (2018)
trust in information source	Experiment 1–3	7-point rating scale	How much do you trust the following sources of health information?<br>example item: TV<br>example item: internet<br>(1 = do not trust at all, 7 = trust completely)	Haase et al. (2015)
frequency of using information source	Experiment 1–3	7-point rating scale	How often do you use the following sources to get health information?<br>example item: TV<br>example item: internet<br>(1 = never, 7 = daily)	Haase et al. (2015)
relevance of source	Experiment 1–3	product score of trust in information source and frequency of using information source		Haase et al. (2015)

**Additional variables**				
willingness to donate	Experiment 1	single item	Please indicate whether you would support the initiative and, if so, how much money you would donate.<br>1 = no support, 2 = 1 (Euro) 3 = 5, 4 = 10, 5 = 20, 6 = other amount	n.a.
speaker evaluation	Experiment 1–3	mean score of 7-point semantic differentials<br>competence (α1denier = .90; α1advocate= .95; α2denier = .89; α2advocate= .87; α3denier = .90; α3advocate = .89)<br><br>character (α1denier = .65; α1advocate= .97; α2denier = .61; α2advocate= .96; α3denier = .63; α3advocate = .96)<br><br>sociability (α1denier = .61; α1advocate= .97; α2denier = .69; α2advocate= .67; α3denier = .68; α3advocate = .74)	Please rate name denier/name advocate.<br>Example item competence: 1. qualified 7. unqualified Example item character: 1. selfish 7. unselfish Example item sociability: 1. friendly 7. unfriendly	McCroseky & Johnson (1975)
attention check 1	Experiment 1–3	Single item selection	What was the TV debate about?<br>(1 = About the effectiveness of the vaccination against dysomeria compared to the vaccination against verococci; 2 = About the vaccination against dysomeria; 3 = About the vaccination record of Stefan Müller. 4 = About the vaccination against verococci.)	n.a.
attention check 2	Experiment 2–3	Open textbox	Please enter the correct number.<br>1. How many guests from [*denier group*] were present in the TV debate?<br>2. How many guests from [*advocate group*] were present in the TV debate?	n.a.

Supplementary Table 1. Overview of additional measures. Reliability of multiple-item scales is indicated by Cronbach’s alpha; numbers behind alphas relate to the respective experiments.

	Experiment 1			Experiment 2			Experiment 3		
Change in Attitude	Mean	*SD*	*n*	Mean	*SD*	*n*	Mean	*SD*	*n*
Overall	-15.26	16.29	101	-11.77	18.70	390	-8.67	16.00	396
Conditions									
Rebuttal & 3:3 & No Forewarning	-14.33	16.23	25	-8.17	20.31	50	-2.38	14.23	49
Rebuttal & 5:1 & No Forewarning	-6.73	15.64	26	-10.20	17.45	49	-5.03	12.24	48
Advocate silent & 3:3 & No Forewarning	-17.33	12.48	25	-17.35	18.62	49	-14.00	14.62	50
Advocate silent & 5:1 & No Forewarning	-23.00	16.89	25	-21.53	18.90	48	-16.17	18.70	50
Rebuttal & 3:3 & Forewarning	--	--	--	-5.21	16.63	48	-4.42	10.91	49
Rebuttal & 5:1 & Forewarning	--	--	--	-5.61	16.61	49	-1.02	12.57	49
Advocate silent & 3:3 & Forewarning	--	--	--	-14.58	19.33	48	-9.31	15.60	51
Advocate silent & 5:1 & Forewarning	--	--	--	-11.73	16.31	49	-16.50	18.93	50
Main effects									
Rebuttal	-10.46	16.23	51	-7.31	17.81	196	-3.21	12.55	195
Advocate silent	-20.17	14.97	50	-16.28	18.53	194	-13.97	17.18	201
3:3	-15.83	14.41	50	-11.32	19.27	195	-7.58	14.58	199
5:1	-14.71	18.10	51	-12.22	18.15	195	-9.77	17.27	197
Forewarning	--	--	--	-9.28	17.59	194	-7.87	15.85	199
No Forewarning	--	--	--	-14.24	19.47	196	-9.48	16.14	197

Supplementary Table 2. Descriptive data for change in attitude independent of condition (overall) and stratified by conditions and experimental groups. Values are presented as percentages of maximum possible scores of the original scales (POMP), with smaller numbers indicating greater influence from science deniers.

	Experiment 1			Experiment 2			Experiment 3		
Change in Intention	Mean	*SD*	*n*	Mean	*SD*	*n*	Mean	*SD*	*n*
Overall	-11.12	17.21	101	-10.67	17.69	390	-7.11	15.87	396
Conditions									
Rebuttal & 3:3 & No Forewarning	-11.80	17.82	25	-9.60	17.69	50	-0.60	8.21	49
Rebuttal & 5:1 & No Forewarning	-4.23	16.81	26	-8.49	17.46	49	-4.99	12.58	48
Advocate silent & 3:3 & No Forewarning	-12.00	13.76	25	-19.24	19.80	49	-13.49	14.71	50
Advocate silent & 5:1 & No Forewarning	-16.73	18.62	25	-18.48	16.61	48	-16.89	18.78	50
Rebuttal & 3:3 & Forewarning	--	--	--	-2.42	10.22	48	-0.16	11.97	49
Rebuttal & 5:1 & Forewarning	--	--	--	-2.80	17.00	49	0.08	12.81	49
Advocate silent & 3:3 & Forewarning	--	--	--	-12.75	17.93	48	-9.29	16.43	51
Advocate silent & 5:1 & Forewarning	--	--	--	-11.65	16.25	49	-11.03	18.98	50
Main effects									
Rebuttal	-7.94	17.56	51	-5.86	16.14	196	-1.40	11.63	195
Advocate silent	-14.36	16.38	50	-15.53	17.89	194	-12.66	17.41	201
3:3	-11.90	15.76	50	-11.03	17.77	195	-5.96	14.33	199
5:1	-10.36	18.64	51	-10.31	17.64	195	-8.28	17.24	197
Forewarning	--	--	--	-7.40	16.27	194	-5.17	16.06	199
No Forewarning	--	--	--	-13.91	18.47	196	-9.08	15.47	197

Supplementary Table 3. Descriptive data for change in intention independent of condition (overall) and stratified by conditions and experimental groups. Values are presented as percentages of maximum possible scores of the original scales (POMP), with smaller numbers indicating greater influence from science deniers.

	Experiment 2			Experiment 3		
Change in Confidence	Mean	*SD*	*n*	Mean	*SD*	*n*
Overall	-8.16	17.56	390	-5.77	15.98	396
Conditions						
Rebuttal & 3:3 & No Forewarning	-2.33	17.10	50	0.68	12.25	49
Rebuttal & 5:1 & No Forewarning	-5.61	16.35	49	-4.17	14.99	48
Advocate silent & 3:3 & No Forewarning	-17.18	15.44	49	-13.50	14.07	50
Advocate silent & 5:1 & No Forewarning	-18.40	17.53	48	-14.50	17.24	50
Rebuttal & 3:3 & Forewarning	1.39	14.92	48	1.36	12.31	49
Rebuttal & 5:1 & Forewarning	-3.57	16.58	49	4.08	14.56	49
Advocate silent & 3:3 & Forewarning	-10.76	16.84	48	-8.66	13.12	51
Advocate silent & 5:1 & Forewarning	-9.01	16.57	49	-10.83	17.44	50
Main effects						
Rebuttal	-2.55	16.33	196	0.51	13.79	195
Advocate silent	-13.83	16.96	194	-11.86	15.62	201
3:3	-7.22	17.55	195	-5.11	14.33	199
5:1	-9.10	17.57	195	-6.43	17.50	197
Forewarning	-5.50	16.81	194	-3.60	15.72	199
No Forewarning	-10.80	17.93	196	-7.95	15.98	197

Supplementary Table 4. Descriptive data for change in confidence independent of condition (overall) and stratified by conditions and experimental groups. Values are presented as percentages of maximum possible scores of the original scales (POMP), with smaller numbers indicating greater influence from science deniers.

Attitude	Experiment 1 n = 98			Experiment 2 n = 389			Experiment 3 n = 382		
Effects	F	p	η^2_p	F	p	η^2_p	F	p	η^2_p
Time	2.70	.104	.030	0.19	.661	.001	**10.08**	**.002**	**.027**
Rebuttal × Time	**7.98**	**.006**	**.083**	**20.73**	**<.001**	**.052**	**47.00**	**<.001**	**.113**
Outnumbering × Time	0.12	.727	.001	0.65	.422	.002	1.46	.228	.004
Forewarning × Time	--	--	--	**7.06**	**.008**	**.018**	2.01	.157	.005
Rebuttal × Outnumbering × Time	**4.99**	**.028**	**.054**	0.02	.880	<.001	2.10	.148	.006
Rebuttal × Forewarning × Time	--	--	--	0.24	.627	.001	0.19	.661	.001
Outnumbering × Forewarning × Time	--	--	--	1.41	.236	.004	0.05	.824	<.001
Rebuttal × Outnumbering × Forewarning × Time	--	--	--	1.20	.273	.003	2.35	.126	.006
Knowledge about vaccination × Time	**5.99**	**.016**	**.064**	**7.14**	**.008**	**.019**	**15.92**	**<.001**	**.041**
Source Relevance Television × Time	0.03	.875	<.001	2.17	.142	.006	1.99	.160	.005
Education low × Time	Ref.	--	--	Ref.	--	--	Ref.	--	--
Education middle × Time	0.02	.888	<.001	0.09	.762	<.001	1.21	.272	.003
Education high × Time	0.10	.759	.001	<0.01	.963	<.001	0.12	.726	**<.001**
Gender male × Time	Ref.	--	--	Ref.	--	--	Ref.	--	--
Gender female × Time	0.12	.733	.001	**4.34**	**.038**	**.011**	0.96	.327	.003
Age × Time	1.64	.204	.018	0.66	.418	.002	2.72	.100	.007

Supplementary Table 5. Effects from rebuttal and weight-of-evidence strategies on changes in attitude after controlling for effects of preregistered covariates. All models are repeated-measures ANOVAs (Type 2 sum of squares) on change scores of individuals' attitudes. Significant effects are shown in boldface for the significance level of <0.05.

Intention	Experiment 1 n = 98			Experiment 2 n = 389			Experiment 3 n = 382		
Effects	F	p	η^2_p	F	p	η^2_p	F	p	η^2_p
Time	1.00	.319	.011	0.48	.488	.001	2.09	.150	.006
Rebuttal × Time	2.50	.117	.028	**29.06**	**<0.01**	**.072**	**62.06**	**<.001**	**.145**
Outnumbering × Time	0.39	.535	.004	0.02	.902	<.001	1.70	.194	.005
Forewarning × Time	--	--	--	**14.04**	**<.001**	**.036**	**10.26**	**.002**	**.027**
Rebuttal × Outnumbering × Time	3.91	.051	.042	0.03	.855	<.001	.001	.979	<.001
Rebuttal × Forewarning × Time	--	--	--	0.03	.864	<.001	0.39	.531	.001
Outnumbering × Forewarning × Time	--	--	--	0.06	.809	<.001	1.55	.215	.004
Rebuttal × Outnumbering × Forewarning × Time	--	--	--	0.29	.592	.001	0.08	.774	<.001
Knowledge about vaccination × Time	2.08	.153	.023	**4.45**	**.035**	**.012**	**7.92**	**.005**	**.021**
Source Relevance Television × Time	0.18	.669	.002	0.86	.355	.002	6.25	.013	.017
Education low × Time	Ref.	--	--	Ref.	--	--	Ref.	--	--
Education middle × Time	0.16	.694	.002	0.32	.569	.001	0.17	.685	<.001
Education high × Time	0.02	.883	<.001	<0.01	.952	<.001	0.03	.873	<.001
Gender male × Time	Ref.	--	--	Ref.	--	--	Ref.	--	--
Gender female × Time	0.13	.716	.002	3.83	.051	.010	**4.41**	**.036**	**.012**
Age × Time	1.89	.172	.027	0.06	.812	<.001	1.01	.317	.003

Supplementary Table 6. Effects from rebuttal and weight-of-evidence strategies on changes in intention after controlling for preregistered covariates' effects. All models are repeated-measures ANOVAs (Type 2 sum of squares) on change scores of individuals' attitudes. Significant effects are shown in boldface for the significance level of <0.05.

Experiment 1	Model 1 Attitude $n = 101$			Model 2 Intention $n = 101$					
	F	*p*	η^2_p	*F*	*p*	η^2_p			
Rebuttal	**10.52**	**.002**	**.100**	**4.47**	**.037**	**.045**			
Outnumbering	0.10	.749	.001	0.22	.639	.002			
Involvement	0.85	.358	.009	0.24	.628	.002			
Rebuttal × Outnumbering	**5.21**	**.025**	**.052**	2.65	.110	.027			
Outnumbering × Involvement	0.52	.472	.005	1.00	.321	.010			
Experiment 2	Model 3 Attitude $n = 390$			Model 4 Intention $n = 390$			Model 5 Confidence $n = 390$		
	F	*p*	η^2_p	*F*	*p*	η^2_p	*F*	*p*	η^2_p
Rebuttal	**24.10**	**<.001**	**.060**	**31.89**	**<.001**	**.078**	**45.70**	**<.001**	**.108**
Outnumbering	0.22	.643	.001	0.15	.699	<.001	1.09	.296	.003
Forewarning	**7.60**	**.006**	**.020**	**14.26**	**<.001**	**.036**	**9.85**	**.002**	**.025**
Involvement	0.05	.824	<.001	0.28	.598	.001	**4.34**	**.038**	**.011**
Rebuttal × Outnumbering	0.02	.898	<.001	0.03	.857	<.001	1.88	.171	.005
Rebuttal × Forewarning	0.44	.509	.001	<0.01	.952	<.001	2.12	.146	.006
Outnumbering × Forewarning	1.45	.229	.004	0.04	.838	<.001	<0.01	.952	<.001
Rebuttal × Outnumbering × Forewarning	0.52	.471	.001	0.07	.791	<.001	0.44	.510	.001
Outnumbering × Involvement	0.58	.448	.002	<0.01	.944	<.001	0.27	.604	.001
Forewarning × Involvement	0.26	.611	.001	<0.01	.958	<.001	1.70	.193	.004
Experiment 3	Model 6 Attitude $n = 396$			Model 7 Intention $n = 396$			Model 8 Confidence $n = 396$		
	F	*p*	η^2_p	*F*	*p*	η^2_p	*F*	*p*	η^2_p
Rebuttal	**50.86**	**<.001**	**.120**	**63.50**	**<.001**	**.144**	**72.10**	**<.001**	**.162**
Outnumbering	1.25	.265	.003	1.42	.234	.004	0.48	.489	.001
Forewarning	2.10	.148	.006	**10.91**	**.001**	**.028**	**10.39**	**.001**	**.027**
Involvement	0.10	.756	<.001	1.24	.266	.003	**4.17**	**.042**	**.011**
Rebuttal × Outnumbering	1.84	.175	.005	0.01	.937	<.001	0.02	.901	<.001
Rebuttal × Forewarning	0.04	.851	<.001	0.30	.587	.001	0.12	.727	<.001

Outnumbering × Forewarning	0.08	.773	<.001	2.62	.106	.007	1.72	.190	.005
Rebuttal × Outnumbering × Forewarning	3.01	.083	.008	0.22	.640	.001	2.31	.129	.006
Outnumbering × Involvement	**5.95**	**.015**	**.016**	2.75	.098	.007	1.32	.252	.004
Forewarning × Involvement	0.02	.881	<.001	0.02	.894	<.001	0.22	.637	.001

Supplementary Table 7. The efficacy of outnumbering and forewarning as a function of the audience's issue involvement. All models are linear models (Type 2 sum of squares) on change scores of the respective outcome measure. Significant effects are shown in boldface for the significance level of <0.05.

Confidence	Experiment 2 n = 389			Experiment 3 n = 382		
Effects	F	p	η^2_p	F	p	η^2_p
Time	**6.24**	**.013**	**.016**	**7.78**	**.006**	**.021**
Rebuttal × Time	**44.75**	**<.001**	**.107**	**68.63**	**<.001**	**.157**
Outnumbering × Time	2.14	.145	.006	0.57	.450	.002
Forewarning × Time	**9.29**	**.002**	**.024**	**11.56**	**.001**	**.030**
Rebuttal × Outnumbering × Time	0.62	.432	.002	0.03	.872	<.001
Rebuttal × Forewarning × Time	1.14	.286	.003	0.15	.698	<.001
Outnumbering × Forewarning × Time	<0.01	.991	<.001	1.49	.224	.004
Rebuttal × Outnumbering × Forewarning × Time	1.25	.263	.003	1.54	.215	.004
Knowledge about vaccination × Time	**12.97**	**<.001**	**.033**	**12.74**	**<.001**	**.033**
Source Relevance Television × Time	0.06	.801	<.001	2.03	.156	.005
Education low × Time	Ref.	--	--	Ref.	--	--
Education middle × Time	0.10	.755	<.001	**4.29**	**.039**	**.012**
Education high × Time	0.01	.920	<.001	2.92	.088	.008
Gender male × Time	Ref.	--	--	Ref.	--	--
Gender female × Time	**5.03**	**.026**	**.013**	0.55	.460	.001
Age × Time	3.25	.072	.009	0.50	.482	.001

Supplementary Table 8. Effects from rebuttal and weight-of-evidence strategies on changes in confidence after controlling for preregistered covariates' effects. All models are repeated-measures ANOVAs (Type 2 sum of squares) on change scores of individuals' attitudes. Significant effects are shown in boldface for the significance level of <0.05.

Attitude	Experiment 2 n = 280			Experiment 3 n = 299		
Effects	F	p	η^2_p	F	p	η^2_p
Time	**127.96**	**<.001**	**.320**	**85.51**	**<.001**	**.227**
Rebuttal × Time	**16.75**	**<.001**	**.058**	**37.23**	**<.001**	**.113**
Outnumbering × Time	0.04	.848	<.001	0.03	.871	<.001
Forewarning × Time	**7.67**	**.006**	**.027**	2.04	.154	.007
Rebuttal × Outnumbering × Time	1.45	.230	.005	0.50	.479	.002
Rebuttal × Forewarning × Time	<0.01	.995	<.001	0.30	.584	.001
Outnumbering × Forewarning × Time	0.58	.446	.002	0.06	.811	<.001
Rebuttal × Outnumbering × Forewarning × Time	0.04	.847	<.001	**4.84**	**.029**	**.016**

Supplementary Table 9. Effects from rebuttal and weight-of-evidence strategies on changes in attitude with a sample containing only those participants who recalled the correct information. All models are repeated-measures ANOVAs (Type 2 sum of squares) on change scores of individuals' attitudes. Significant effects are shown in boldface for the significance level of <0.05.

Intention	Experiment 2 n = 280			Experiment 3 n = 299		
Effects	F	p	η^2_p	F	p	η^2_p
Time	**127.48**	**<.001**	**.319**	**52.26**	**<.001**	**.152**
Rebuttal × Time	**26.11**	**<.001**	**.088**	**37.08**	**<.001**	**.113**
Outnumbering × Time	1.14	.286	.004	0.30	.586	.001
Forewarning × Time	**18.73**	**<.001**	**.064**	**5.45**	**.020**	**.018**
Rebuttal × Outnumbering × Time	0.35	.555	.001	1.14	.286	.004
Rebuttal × Forewarning × Time	<0.00	.956	<.001	0.05	.827	<.001
Outnumbering × Forewarning × Time	0.10	.749	<.001	0.03	.853	**<.001**
Rebuttal × Outnumbering × Forewarning × Time	0.04	.852	<.001	2.38	.124	.008

Supplementary Table 10. Effects from rebuttal and weight-of-evidence strategies on changes in intention with a sample containing only those participants who recalled the correct information. All models are repeated-measures ANOVAs (Type 2 sum of squares) on change scores of individuals' attitudes. Significant effects are shown in boldface for the significance level of <0.05.

Confidence	Experiment 2 n = 280			Experiment 3 n = 299		
Effects	F	p	η^2_p	F	p	η^2_p
Time	**68.51**	**<.001**	**.201**	**24.04**	**<.001**	**.076**
Rebuttal × Time	**43.57**	**<.001**	**.138**	**58.10**	**<.001**	**.166**
Outnumbering × Time	0.16	.685	.001	0.02	.877	<.001
Forewarning × Time	**4.53**	**.034**	**.016**	**7.64**	**.006**	**.026**
Rebuttal × Outnumbering × Time	1.72	.191	.006	0.01	.910	<.001
Rebuttal × Forewarning × Time	0.81	.369	.003	0.57	.451	.002
Outnumbering × Forewarning × Time	0.16	.688	.001	0.02	.888	<.001
Rebuttal × Outnumbering × Forewarning × Time	0.02	.885	<.001	**7.04**	**.008**	**.024**

Supplementary Table 11. Effects from rebuttal and weight-of-evidence strategies on changes in confidence with a sample containing only those participants who recalled the correct information. All models are repeated-measures ANOVAs (Type 2 sum of squares) on change scores of individuals' attitudes. Significant effects are shown in boldface for the significance level of <0.05.

Post values Attitude	Experiment 3 n = 387		
Effects	F	p	η^2_p
Intercept	**77.65**	**<.001**	**.616**
Prior Attitude	**563.18**	**<.001**	**.593**
Rebuttal	**52.02**	**<.001**	**.118**
Outnumbering	1.83	.177	.005
Forewarning	1.16	.282	.003
Rebuttal × Outnumbering	2.63	.106	.007
Rebuttal × Forewarning	0.20	.652	.001
Outnumbering × Forewarning	0.15	.704	<.001
Rebuttal × Outnumbering × Forewarning	2.25	.135	.006

Supplementary Table 12. Effects from rebuttal and weight-of-evidence strategies on post-attitude values controlled for pre-attitude values. The model is a repeated-measures ANOVA (Type 2 sum of squares) on change scores of individuals' attitudes. Significant effects are shown in boldface for the significance level of <0.05.

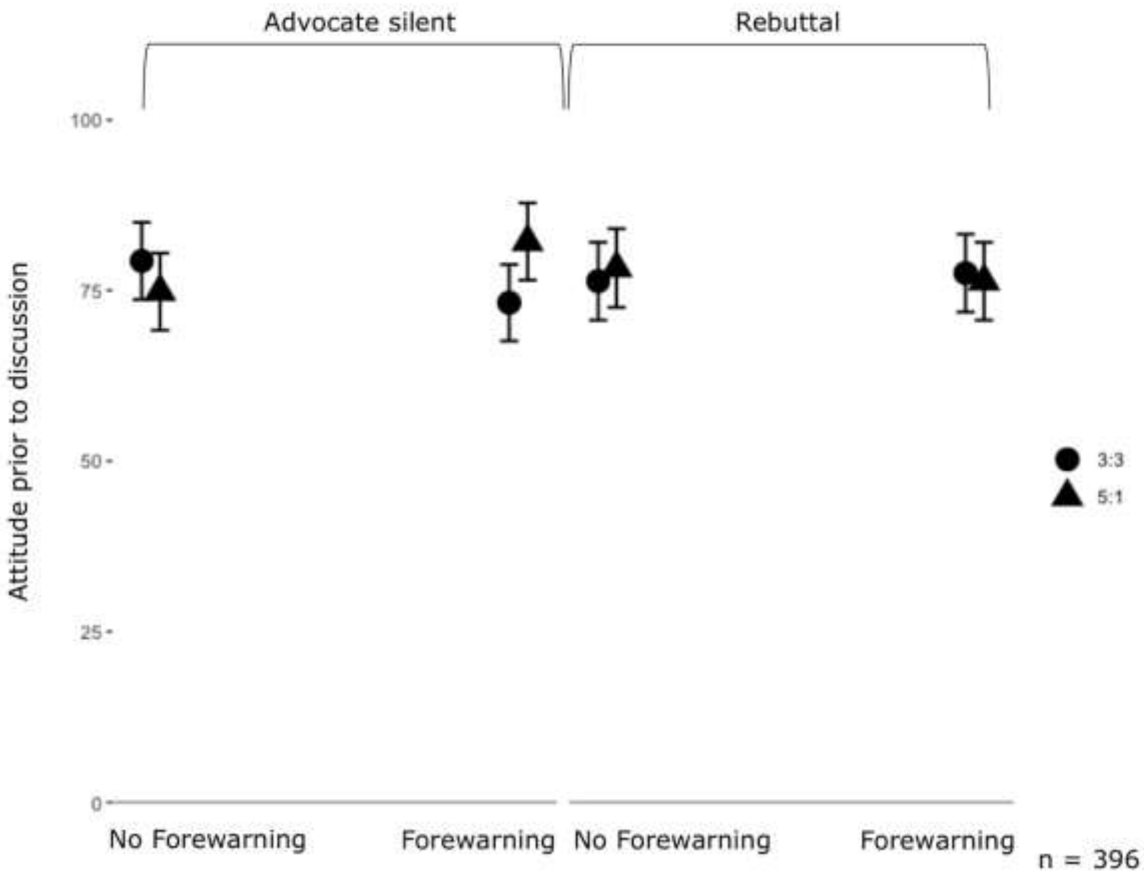

Supplementary Figure 1. Evidence of a biased distribution of pre-values in Experiment 3. The figure visualises a three-way interaction of rebuttal × outnumbering × forewarning in an ANOVA model on individuals' attitude values prior to the discussion. Circles and triangles represent mean values. Error bars are 95% confidence intervals. Results from ANOVAs stratified by rebuttal revealed that the three-way interaction resulted from a significant two-way interaction from outnumbering × forewarning on individuals' attitudes in the advocate-silent condition, $F(1, 197) = 5.92$, $p = .016$, $\eta^2_p = .029$, and a reversed, but insignificant, pattern in the rebuttal condition, $F(1, 191) = 0.27$, $p = .607$, $\eta^2_p = .001$. Further exploration of simple effects in the advocate-silent condition revealed that the two-way interaction resulted from significantly lower pre-values of participants in the 5:1 condition (outnumbering) compared with participants in the 3:3 condition, $p = .028$, when participants were forewarned and a reversed, but insignificant, pattern in the no-forewarning condition, $p = .271$.

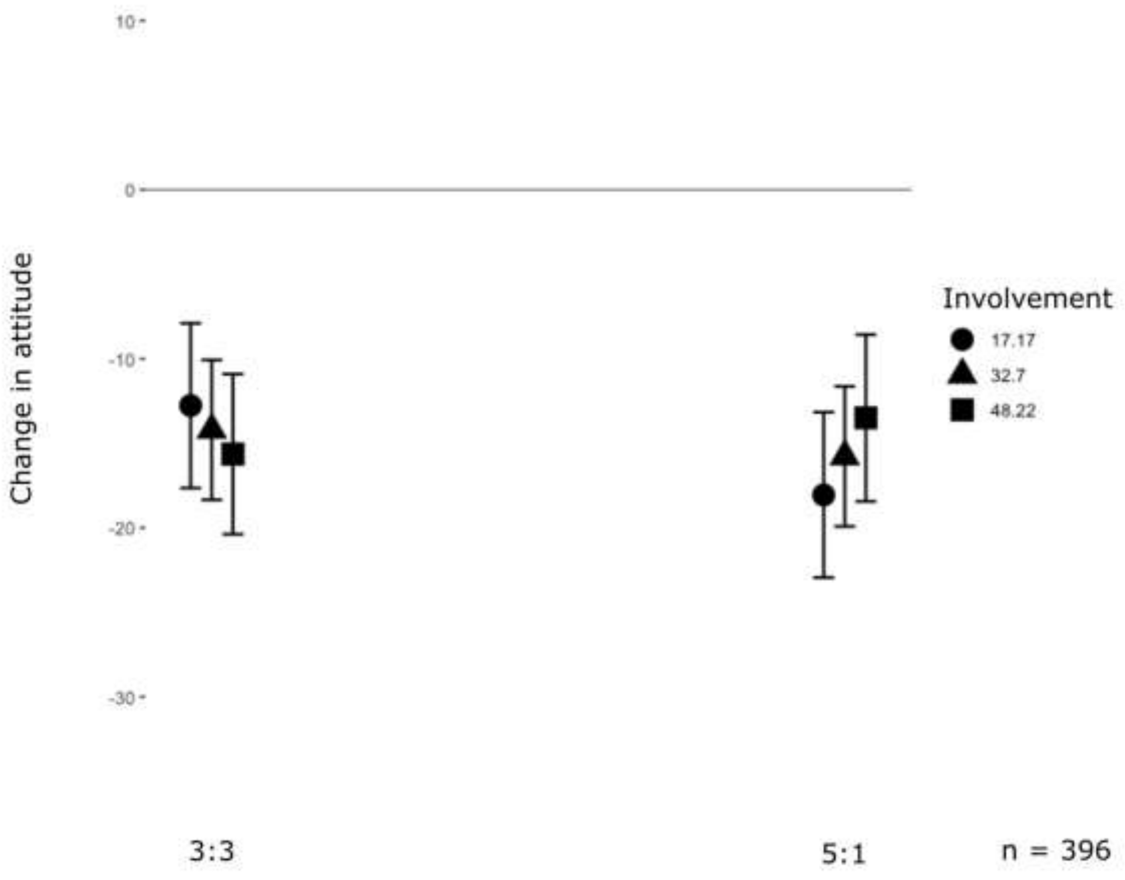

Supplementary Figure 2. Outnumbering increases damage from denialism with decreasing issue involvement from the audience. The figure visualises a two-way interaction of outnumbering × involvement in a linear model (Supplementary Table 7) on changes in individuals' attitude values in Experiment 3. Circles and triangles represent mean values. Error bars are 95% confidence intervals. Results on attitude changes for moderator levels stratified by outnumbering revealed that the damage from deniers increases with decreasing issue involvement, but only if the denier was outnumbered (5:1). The pattern is reversed for the falsely balanced discussion. The pattern contradicts a conditional benefit from outnumbering (5:1) compared with a falsely balanced discussion (3:3), as expected by previous research underlying the involvement as a moderator research question.

Supplementary References

Zaichkowsky, J. L. (1985). Measuring the involvement construct. *Journal of consumer research*, *12*(3), 341-352.

Zingg, A., & Siegrist, M. (2012). Measuring people's knowledge about vaccination: developing a one-dimensional scale. *Vaccine*, *30*(25), 3771-3777.

Betsch, C., Schmid, P., Heinemeier, D., Korn, L., Holtmann, C., & Böhm, R. (2018). Beyond confidence: Development of a measure assessing the 5C psychological antecedents of vaccination. *PloS one*, *13*(12), e0208601.

Haase, N., Betsch, C., & Renkewitz, F. (2015). Source credibility and the biasing effect of narrative information on the perception of vaccination risks. *Journal of health communication*, *20*(8), 920-929.

McCroskey, J. C., & Jenson, T. A. (1975). Image of mass media news sources. *Journal of Broadcasting & Electronic Media*, *19*(2), 169-180.
